# Structure–Activity Relationship Analysis of Rhosin, a RhoA GTPase Inhibitor, Reveals a New Class of Antiplatelet Agents

**DOI:** 10.3390/ijms24044167

**Published:** 2023-02-19

**Authors:** Akhila Dandamudi, William Seibel, Benjamin Tourdot, Jose A. Cancelas, Huzoor Akbar, Yi Zheng

**Affiliations:** 1 Division of Experimental Hematology and Cancer Biology, Cincinnati Children’s Hospital Medical Center, 3333 Burnet Avenue, Cincinnati, OH 45229, USA; 2 Department of Pathology, University of Cincinnati Graduate School, Cincinnati, OH 45267, USA; 3 Division of Oncology, Cincinnati Children’s Hospital Medical Center, University of Cincinnati, 3333 Burnet Avenue, Cincinnati, OH 45229, USA; 4 Hoxworth Blood Center, College of Medicine, University of Cincinnati, Cincinnati, OH 45229, USA; 5 Department of Biomedical Sciences, Heritage College of Osteopathic Medicine, Ohio University, Athens, OH 45701, USA

**Keywords:** small-molecule inhibitor, chiral enantiomer, Rho GTPase signaling, platelet activation, antiplatelet, thrombosis, hemostasis

## Abstract

Current antiplatelet therapies have several clinical complications and are mostly irreversible in terms of suppressing platelet activity; hence, there is a need to develop improved therapeutic agents. Previous studies have implicated RhoA in platelet activation. Here, we further characterized the lead RhoA inhibitor, Rhosin/G04, in platelet function and present structure–activity relationship (SAR) analysis. A screening for Rhosin/G04 analogs in our chemical library by similarity and substructure searches revealed compounds that showed enhanced antiplatelet activity and suppressed RhoA activity and signaling. A screening for Rhosin/G04 analogs in our chemical library using similarity and substructure searches revealed compounds that showed enhanced antiplatelet activity and suppressed RhoA activity and signaling. SAR analysis revealed that the active compounds have a quinoline group optimally attached to the hydrazine at the 4-position and halogen substituents at the 7- or 8-position. Having indole, methylphenyl, or dichloro-phenyl substituents led to better potency. Rhosin/G04 contains a pair of enantiomers, and S-G04 is significantly more potent than R-G04 in inhibiting RhoA activation and platelet aggregation. Furthermore, the inhibitory effect is reversible, and S-G04 is capable of inhibiting diverse-agonist-stimulated platelet activation. This study identified a new generation of small-molecule RhoA inhibitors, including an enantiomer capable of broadly and reversibly modulating platelet activity.

## 1. Introduction

Platelets are small, anucleated cellular fragments released by bone marrow precursors, megakaryocytes. The primary role of platelets is to maintain hemostasis by serving as sentinels of vascular integrity. Platelets encounter the subendothelial extracellular matrix upon vascular damage and undergo adhesion, shape change, granular secretion, and aggregation, resulting in thrombus formation [1]. Highly reactive platelets in diabetes, hypertension, or plaque rupture in atherosclerotic patients contribute to pathologic thrombosis, leading to life-threatening cardiovascular complications such as myocardial infarction and stroke [2,3,4].

Due to the vital role of activated platelets in the development of cardiovascular diseases, antiplatelet agents such as aspirin and clopidogrel are widely prescribed to prevent and manage thrombotic complications in high-risk individuals. Aspirin was the first and most commonly used antiplatelet medication and is a milestone in the modern pharmaceutical industry [5]. Dual antiplatelet therapy (DAPT) with aspirin and clopidogrel is the standard of care to reduce the recurrence of major cardiovascular events in patients, but this causes an increased risk of bleeding compared with aspirin monotherapy [6]. Newer and more potent FDA-approved antiplatelet agents include thienopyridines (ticlopidine, clopidogrel, and prasugrel), abciximab, cilostazol, dipyridamole, tirofiban, and eptifibatide [7,8,9]. However, many of the current antiplatelet drugs pose several clinical complications, such as increased risk of bleeding, uncontrolled thrombosis, significant inter-individual variability, narrow therapeutic window, and an irreversible mechanism of action, thus affecting normal hemostasis if the need for emergency surgery arises [10,11]. Hence, it is essential to identify improved antiplatelet therapies that are safer and more effective.

Rho family GTPases such as RhoA, Rac1, and Cdc42 have been implicated in platelet activation pathways such as the regulation of platelet spreading [12], retraction [13], secretion [14,15,16,17], and aggregation [14,15,16,17,18]. As most canonical Rho GTPases, RhoA cycles between the inactive GDP-bound and active GTP-bound forms. Active RhoA binds to the downstream effector Rho-associated coiled-coil-containing protein kinase (ROCK), which mediates the phosphorylation of the myosin light chain (MLC) via the G_α13_/RhoA/ROCK pathway, leading to platelet shape change and secretion [19,20,21]. Previous studies have shown that targeting RhoA blocks the activation of ROCK and ROS production [22], highlighting the potential of RhoA as a target for inhibiting platelet activation.

Previously, we discovered a RhoA-specific inhibitor, Rhosin, also termed G04, based on the structure–function relationship between RhoA interaction and its activator, guanine nucleotide exchange factor (GEF) [23]. Rhosin/G04 contains two aromatic rings (a quinoxaline and an indole) tethered by a “flexible” linker that is believed to bind to the Trp58 region of the RhoA surface required for GEF interaction with micromolar affinity (Supporting information Figure S1) [23,24]. Rhosin inhibits platelet spreading on fibrinogen and thrombin-induced platelet aggregation, mimicking the effects of *RhoA* genetic deletion [25]. While Rhosin/G04 is a promising proof-of-concept compound, it is not very potent and has a poor pharmacokinetic profile (unpublished data), highlighting the need for improvement. In addition, several studies have highlighted that hydrazones, hydrazines, and indole moieties can serve as essential pharmacophores for inhibiting platelet activation [26,27,28,29,30,31,32,33]. In the current work, we examine analogs of Rhosin/G04 in inhibiting platelet activation and RhoA activity and report the structure–activity relationship (SAR) and the discovery of G04 chiral enantiomers, which may lead to a new class of antiplatelet agents.

## 2. Results and Discussion

Rhosin/G04 was identified by means of the RhoA-targeted docking of more than four million compounds from an in silico compound library obtained with the ZINC purchasable compound set (International Zinc Association, Washington, DC) [23,34]. Rhosin/G04 contains two aromatic rings tethered by a linker that is believed to bind to the Trp58 region of the RhoA surface required for GEF interaction with micromolar affinity [23,24]. Rhosin/G04 can be synthesized by means of the formal condensation of the carboxy group of D-tryptophan with the amino group of (quinoxalin-6-yl)methylidenehydrazide [35]. Based on the findings obtained by Shang et al. [23], we believe that the preliminary pharmacophore consisting of an Indole/Benzimidazole ring tethered to a quinoxaline through a flexible linker of four–eight atoms retains inhibitory activity against RhoA. The University of Cincinnati (UC)/Cincinnati Children’s Hospital Medical Center (CCHMC) compound library of over 360,000 compounds was filtered using a series of substructure and similarity searches for compounds broadly related to this pharmacophore. Seven compounds were selected as they are diverse, show plausible binding pose and score, adhere to the preliminary pharmacophore, and avoid problematic functionality (pan-assay interference compounds (PAINSs), reactive/unstable, or toxicology-linked).

Light transmission aggregometry, i.e., the Born method, was used to determine in vitro antiplatelet aggregation activity using collagen as the agonist for platelet aggregation [36]. Collagen was selected as the agonist, since it is the most thrombogenic component of the subendothelial layer [37]. Collagen also serves as a substrate for platelet adhesion and as an inducer of platelet activation [38,39]. The inhibitory potential of these seven compounds was assessed using a low collagen concentration (1 µg/mL) and a high collagen concentration (5 µg/mL), since cyclooxygenase (COX) inhibitors impact low-collagen-induced aggregation but higher concentrations are unaffected [40,41]. Indomethacin (20 µM), a reversible COX-1 inhibitor, and cangrelor (20 µM), a reversible P_2_Y_12_ inhibitor, were chosen as the positive controls to compare the anti-aggregation effect of Rhosin analogs [42,43,44]. UC-177629 emerged as the only potent inhibitor of collagen-induced platelet aggregation in washed human platelets (Table 1). Thirteen analogs of UC-177629 were also evaluated in the platelet aggregation assay at low and high concentrations of collagen, with eight of the compounds showing good activity and five compounds showing minimal activity. These analogs and ten additional compounds that are less closely related but have functionality that might improve physical properties were evaluated; however, the activity was not as compelling (Table 2). Therefore, the top 12 most active compounds that inhibited collagen-induced aggregation by at least 70% in washed human platelets were selected for subsequent analysis.

**Figure 1:**
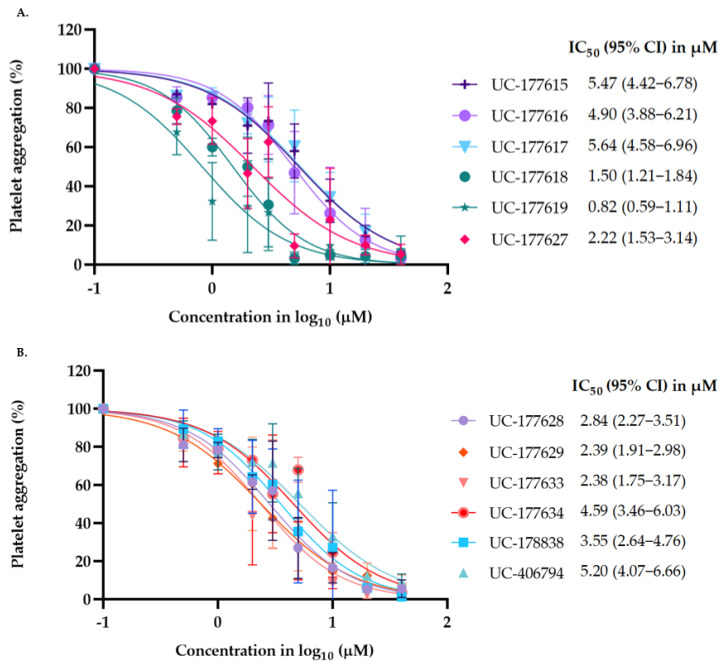
Half-maximal inhibitory concentrations (IC_50_) of Rhosin-related compounds. (**A**,**B**) The IC_50_ and 95% CI values of active Rhosin-related compounds were calculated using the dose–response curves for washed human platelets using collagen (1 µg/mL) as an inducer of platelet aggregation. Data are representative of three independent experiments.

The cytotoxicity of the active analogs was determined with the lactate dehydrogenase (LDH) leakage assay to ensure that platelet inhibition was a consequence of pharmacologic inhibition and not cytotoxicity. LDH is a stable cytosolic marker for membrane perturbation and is present in many cell types, including platelets [45,46]. The results revealed that none of the 12 analogs differed significantly from DMSO (dimethyl sulfoxide)-treated platelets (Supporting Information Figure S2). Next, dose–response curves for platelet aggregation using collagen (1 µg/mL) as the agonist were used to determine the half-maximal inhibitory concentration (IC_50_) of the 12 active compounds. The IC_50_ and 95% CI (confidence interval) values of the active analogs ranged from 0.8 µM to 5.6 µM (Figure 1).

The inhibitory potential of the analogs that were not subjected to IC_50_ value determination was estimated based on the percent inhibition observed at 20 µM. The SAR within this series was clear and specific, as follows: (1) The quinoline moiety is optimally attached to the hydrazine at the 4-position. (2) Chloro- and trifluoromethyl substituents at the 7- or 8-position improved activity vs. unsubstituted quinolines. (3) Comparable potency was seen in the indole and methylphenyl substituents, with slightly better potency having been observed in the dichloro-phenyl group (UC-177619). (4) Replacement of the quinoline moiety with an indane (UC-178838) surprisingly afforded a nearly equipotent compound, while a positional isomer (e.g., UC-177630) lost all activity (Table 3). Analogs UC-177618, UC-177619, UC-177628, UC-177629, UC-177633, and UC-177634 were selected for subsequent analysis due to their potency.

To confirm the structures of the six most active analogs, they were subjected to ultra-high-performance liquid chromatography–high-resolution mass spectrometry (UHPLC-HRMS). UHPLC-HRMS was performed to determine the accurate mass-to-charge ratio (*m*/*z*), isotope/adduct ion patterns, and fragmentation patterns. No significant degradation product was present, and the results of the six active analogs are shown in Supporting Information Figure S3. The high-resolution mass spectrum of the peak and the fragmentation pattern of the peak at RT = 6.42 min confirmed UC-177618; at RT = 4.82 min, they confirmed UC-177619; at RT = 3.79, they confirmed UC-177628; at RT = 3.18, they confirmed UC-177629; at RT = 3.14, they confirmed UC-177633; and at RT = 2.68, they confirmed UC-177634.

Several studies reported that N-acylheteroaryl hydrazones (NAHs) show efficacy as platelet aggregation inhibitors. A study by Chelucci et al. reported that nonsteroidal anti-inflammatory drugs (NSAIDs) containing the NAH moiety might exhibit better antiplatelet and antithrombotic activity than aspirin [27]. Another study showed that the substituent on the phenyl ring of the NAH subunit might be responsible for improved antiplatelet activity in the presence of multiple agonists, such as arachidonic acid (AA), collagen, and adenosine diphosphate (ADP) [47]. Fraga et al. showed that acylhydrazone derivatives had inhibitory effects on platelet-activating factor (PAF) and presented a significant antithrombotic profile [48]. In silico, in vitro, and in vivo investigations conducted by Khalid et al. showed that a series of hydrazone and sulfonamide derivatives inhibited AA-, ADP-, and collagen-induced platelet aggregation [49]. Researchers at LASSBio have published several papers showing that hydrazine and acylhydrazone moieties are the essential pharmacophoric cores for several antiplatelets, NSAIDs, and analgesic derivatives due to their inhibitory effects on COX and/or 5-lipooxygenase (LOX) [26,29,31]. The same group also reported that arylsulfonate–acylhydrazone derivatives could simultaneously inhibit platelet aggregation induced by collagen, AA, and thrombin [50].

In addition to hydrazone, the Indole ring is also considered an essential structural moiety with antiplatelet activity. Park et al. analyzed the antiplatelet activity of Indole-3-carbinol, an autolysis product of cruciferous vegetables. They found that Indole-3-carbinol could be a potential antithrombotic and antiplatelet agent, since it inhibited collagen-induced platelet aggregation [32]. Mashayekhi et al. synthesized a series of indole hydrazine derivatives and tested their anti-aggregation ability. They found that these compounds inhibited ADP- and AA-induced platelet aggregation [30]. Akhlaghi et al. synthesized N-aryl methyl-substituted Indole derivatives and showed that these compounds effectively inhibited ADP- and AA-induced platelet aggregation [51]. Tavili et al. synthesized and evaluated a series of N1-substituted indole hydrazones and found that they inhibited AA-induced platelet aggregation. Their SAR analysis revealed that lipophilicity of the para-substituted phenyl ring contributed to the increase in the antiplatelet activity of the derivatives [33]. Tehrani et al. showed that the compounds that share a strongly preserved structural backbone, two (hetero) aromatic ring systems linked by a hydrazone bond, inhibited aggregation induced by AA and ADP [28]. Based on the evidence from these studies, it is likely that the antiplatelet activity of these individual pharmacophoric components might be related to RhoA inhibition.

Previously, we showed that removing Rhosin by washing reversed its inhibitory effects on NIH 3T3 cells [23]. The reversible inhibition of Rhosin in collagen-induced platelet aggregation was assessed with similar wash-out experiments, and this reversible nature of Rhosin makes it a valuable antiplatelet drug candidate (Supporting Information Figure S4A,B). After confirming the structures of the above six active analogs, similar wash-out experiments were performed to compare the reversible function of the active analogs with Rhosin reversibility. Supporting Information Figure S4C,D shows the representative aggregation tracings for UC-177618 and UC-177619, confirming that similar to those of Rhosin/G04, the inhibitory effects of these Rhosin analogs are entirely reversible.

**Figure 2:**
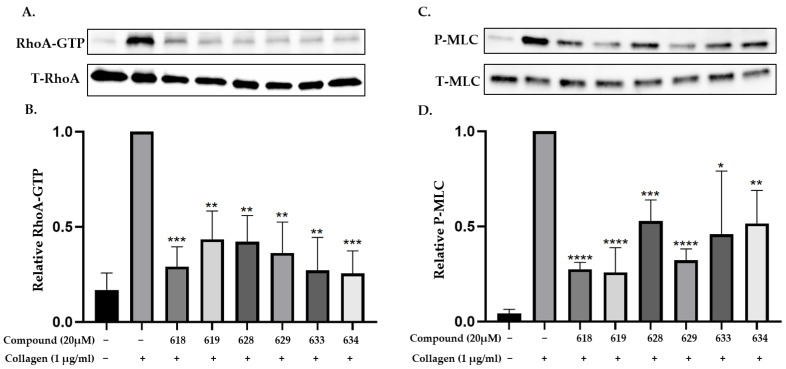
Active Rhosin-related compounds inhibit the activation of RhoA and phosphorylation of the myosin light chain. (**A**,**B**) Washed human platelets were incubated with collagen (1 µg/mL) for 4 min, and the levels of RhoA-GTP formation were quantified with a GST-Rhotekin effector pull-down assay. The platelets were pre-incubated with 20 µM UC-177618, UC-177619, UC-177628, UC-177629, UC-177633, or UC-177633 for 2 min before being stimulated with collagen. The addition of ice-cold lysis buffer terminated the reaction. The top 6 active Rhosin-related compounds inhibited RhoA-GTP formation, and the total cell lysates were also blotted in parallel. Relative amounts of the GTP-bound form of RhoA were quantified with densitometry measurements of total RhoA (T-RhoA) and normalized to untreated platelets. (**C**,**D**) The addition of 20 µM of active Rhosin-related compounds to washed human platelets 2 min before stimulation with collagen blocked the phosphorylation of the myosin light chain (p-MLC). The reactions were terminated after 4 min by adding 4x Laemmli sample buffer. The samples were processed for Western blotting and probed for MLC and p-MLC, and phosphorylation was quantified by measuring the densitometry of total MLC (T-MLC). The differences between DMSO- and Rhosin-related compounds were analyzed with *t*-test, and the results are shown as means ± SDs of three independent experiments. (* *p* < 0.05, ** *p* < 0.01, *** *p* < 0.001, and **** *p* < 0.0001).

**Figure 3:**
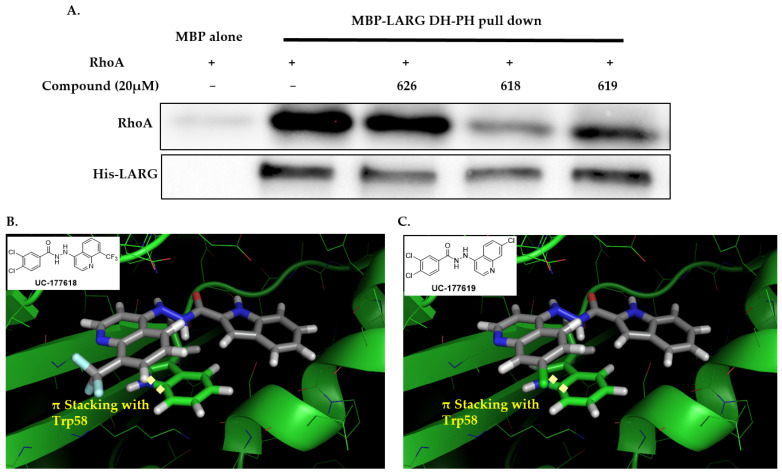
UC-177618 and UC-177619 inhibit RhoA-LARG interaction. (**A**) A complex-formation assay tested the inhibitory effects of 20 µM UC-177618 and UC-177619 on RhoA interaction with the LARG DH-PH domain. MBP-(His)6-tagged LARG (100 ng) was immobilized on Amylose beads, to which binding buffer containing full-length RhoA (100 ng) preincubated with UC-177618, UC-177619, or UC-177626 (an inactive Rhosin-related compound) was added. After incubating the compound-RhoA-LARG mixture at 4 °C for 1 h, the beads associated with RhoA were detected with anti-RhoA Western blotting. (**B**,**C**) Top view of the predicted structural contacts of UC-177618 and UC-177619 in the binding pocket around the Trp58 residue of RhoA.

Next, the ability of the six compounds to suppress RhoA activity in platelets was assessed by subjecting them to a GST (Glutathione-S-transferase)-Rhotekin effector pull-down assay. The compounds were incubated with washed human platelets for 2 min before being stimulated with 1 µg/mL collagen. The analogs inhibited the activation of RhoA at the 20 µM concentration (Figure 2A,B). The analogs also inhibited the phosphorylation of its downstream effector, the MLC (Figure 2C,D). After confirming that these analogs could prevent RhoA activation and the phosphorylation of the MLC, their ability to disrupt complex-formation between RhoA and LARG was tested. The analogs at 20 µM suppressed LARG binding to RhoA under pull-down assay conditions, while inactive analog UC-177626 showed no effects (Figure 3A). Docking analysis (based on PDB ID 5C4M) was used to demonstrate the binding of the top two active analogs, UC-177618 and UC-177619, to RhoA to show that the quinolines of these compounds formed pi-stacking with the Trp58 residue (Figure 3B). These findings indicate that the active analogs inhibit RhoA activation by disrupting the binding between RhoA and its GEF.

Rhosin/G04 is probably derived from R-tryptophan with E geometry across the C=N bond, i.e., Rhosin is the R enantiomer (R-G04). The natural tryptophan enantiomer of G04, i.e., S-G04, was also evaluated. The cytotoxicity of both R-G04 and S-G04 at 50 µM was evaluated in washed human platelets using the LDH assay. As shown in Supporting Information Figure S5, neither R-G04 nor S-G04 induced significant human platelet cytolysis compared with the positive control, membrane detergent Triton X-100. After assessing the non-cytotoxicity of these enantiomers, UHPLC-HRMS was performed, and no significant degradation products were observed. The high-resolution mass spectrum of the peak and the fragmentation pattern of the peak at RT = 1.83 min confirmed R-G04, while the high-resolution mass spectrum of the peak and the fragmentation pattern of the peak at RT = 1.85 min confirmed S-G04 (Supporting Information Figure S6).

**Figure 4:**
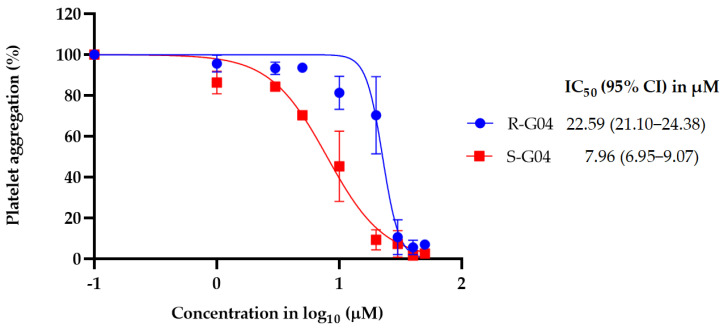
The IC_50_ and 95% CI values for enantiomers R-G04 and S-G04 were calculated using the dose–response curves for washed human platelets using collagen (1 µg/mL) as an inducer of platelet aggregation. Data are representative of three independent experiments.

**Figure 5:**
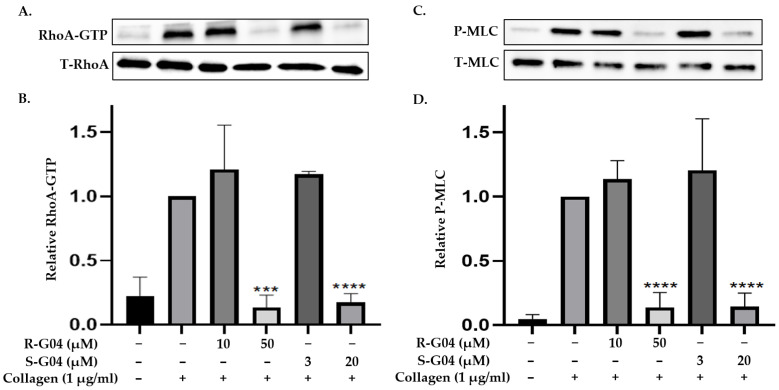
R-G04 and S-G04 inhibit the activation of RhoA and phosphorylation of the myosin light chain**.** (**A**,**B**) Washed human platelets were incubated with collagen (1 µg/mL) for 4 min, and the levels of RhoA-GTP formation were quantified with GST-Rhotekin effector pull-down assay. Platelets were pre-incubated with R-G04 (10 µM or 50 µM) or S-G04 (3 µM or 20 µM) for 2 min before being stimulated with collagen, and the addition of ice-cold lysis buffer terminated the reaction. Both R-G04 and S-G04 inhibited RhoA-GTP formation in a concentration-dependent manner. The total cell lysates were also blotted in parallel. Relative amounts of the GTP-bound form of RhoA were quantified with densitometry measurements of total RhoA (T-RhoA) and normalized to untreated platelets. (**C**,**D**) The addition of different concentrations of R-G04 or S-G04 to washed human platelets 2 min before stimulation with collagen (1 µg/mL) blocked the phosphorylation of the myosin light chain (MLC). The reactions were terminated after 4 min by adding 4x Laemmli sample buffer. The samples were processed for Western blotting and probed for MLC and p-MLC, and phosphorylation was quantified by measuring the densitometry of total MLC (T-MLC). The differences among the DMSO, R-G04, and S-G04 were analyzed using *t*-test, and the results are shown as means ± SDs of three independent experiments. (*** *p* < 0.001 and **** *p* < 0.0001).

S-G04 appeared to be better at inhibiting collagen-induced platelet aggregation than the standard drugs (Table 4). Hence, further antiplatelet analysis of G04 stereoisomers was performed using concentration–response curves using collagen (1 µg/mL) as the agonist. The collagen-induced platelet aggregation assay showed that the IC_50_ value of S-G04 is 7.80 µM, which makes it three times more potent than R-G04 (Figure 4). To examine the specificity of R-G04 and S-G04 in suppressing RhoA activity in platelets, platelets were pre-incubated with different concentrations of R-G04 or S-G04 before being subjected to the GST-Rhotekin effector pull-down assay. As shown in Figure 5B, both R-G04 and S-G04 inhibited RhoA-GTP formation in a concentration-dependent manner. Furthermore, both enantiomers of G04 also inhibited the downstream phosphorylation of the MLC, which is mediated by RhoA effector ROCK (Figure 5C,D). Our findings are further corroborated by Shang et al. [23] and Huzoor et al. [25], who also showed that Rhosin/G04 specifically inhibits the activation of RhoA but not that of Rac1 and Cdc42.

**Figure 6:**
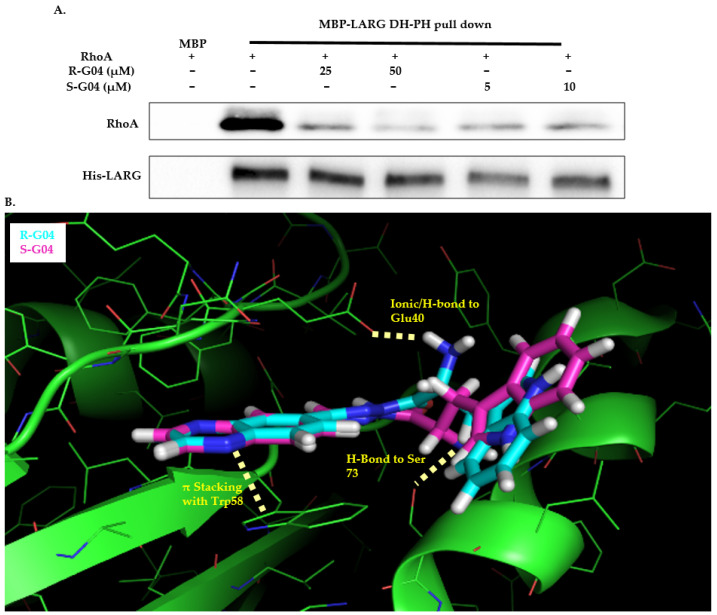
R-G04 and S-G04 inhibit RhoA-LARG interaction. (**A**) A complex-formation assay tested the inhibitory effects of R-G04 and S-G04 on RhoA interaction with the LARG DH-PH domain. MBP-(His)6-tagged LARG (100 ng) was immobilized on Amylose beads. Binding buffer containing full-length RhoA (100 ng) was pre-incubated with different concentrations of R-G04 (25 µM or 50 µM), and S-G04 (5 µM or 10 µM) was added to the beads. After incubating the compound-RhoA-LARG mixture at 4 °C for 1 h, the beads associated with RhoA were detected with anti-RhoA Western blotting. (**B**) Top view of the predicted structural contacts of R-G04 and S-G04 in the binding pocket around the Trp58 residue of RhoA.

To confirm the mechanism of inhibition of G04 enantiomers, a complex-formation assay with RhoA and the LARG DH-PH module was performed by incubating them with RhoA. Both R-G04 and S-G04 were capable of dose-dependently suppressing LARG binding to RhoA under pull-down assay conditions (Figure 6A). Figure 6B shows a hypothetical docking model of R-G04 (cyan) and S-G04 (magenta) in RhoA. RhoA is a flexible protein, particularly across the Switch 1 and Switch 2 regions, which are involved in G04 binding and GEF interaction [23,52]. Docking studies (based on PDB ID 5C4M) were used to depict that the R and S enantiomers of G04 bind similarly, with the quinoxaline anchored with a pi-stacking interaction to Trp-58 within a lipophilic pocket and the indole across Leu-69 and Leu-72, respectively. The amine group of R-G04 formed a hydrogen bond with the Glu-40 residue (3.1 Å), while the amine of S-G04 formed a hydrogen bond with the Ser-73 residue (2.9 Å), which may account for the relatively small three-fold difference in the potency of these compounds. These findings are consistent with our previous work, which found that Rhosin/R-G04 fails to bind to RhoA point mutants bearing Ala mutations around the GEF recognition site [23].

**Figure 7:**
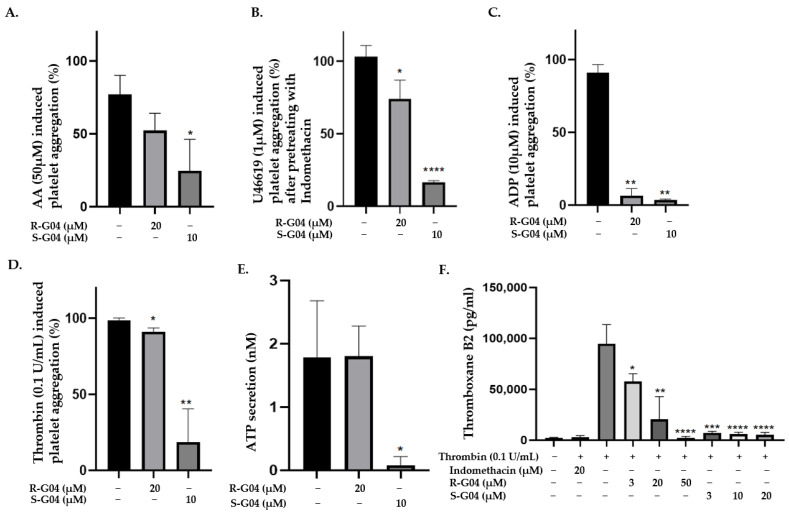
S-G04, but not R-G04, significantly inhibits Arachidonic Acid-, U46619-, and Thrombin-induced platelet aggregation and ATP secretion, while both R-G04 and S-G04 inhibit ADP-induced platelet aggregation and Thromboxane B2 (TxB2) secretion. (**A**–**D**) Washed human platelets were pre-incubated with 10 µM S-G04 or 20 µM R-G04 for 2 min before being stimulated with Arachidonic Acid (AA), U46619, ADP, or Thrombin. The % platelet aggregation was measured 5 min after agonist stimulation. (**E**) Washed human platelets were pre-incubated with 10 µM S-G04 or 20 µM R-G04 for 2 min and with Chrono-lume^®^ for 30 s at 37 °C before being stimulated with 0.1 U/mL Thrombin. (**F**) Washed human platelets were pre-incubated with different concentrations of R-G04 or S-G04 or with 20 µM Indomethacin for 2 min before being stimulated with Thrombin. TxB2 formation was determined using a TxB2 ELISA kit. The differences among DMSO, R-G04, and S-G04 were analyzed using *t*-test, and the results are shown as means ± SDs of three independent experiments. (* *p* < 0.05, ** *p* < 0.01, *** *p* < 0.001, and **** *p* < 0.0001).

After identifying an enantiomer with significantly higher potency than the original R-G04, we applied the enantiomers to antiplatelet assays for inhibiting platelet aggregation induced by multiple agonists, including AA, ADP, U46619, a stable synthetic analog of thromboxane A_2_ (TxA_2_), and thrombin. As shown in Figure 7A–D, 10 µM S-G04 suppressed the platelet activation induced by AA, U46619, and thrombin, and it also inhibited ATP secretion when stimulated with thrombin. Pre-incubating platelets with 20 µM R-G04 failed to have the same inhibitory effect. However, both R-G04 (20 µM) and S-G04 (10 µM) significantly inhibited ADP-induced platelet aggregation. In addition, R-G04 and S-G04 exhibited dose-dependent inhibition on the generation of TxA_2_, measured by means of its stable metabolite, thromboxane B_2_ (TxB_2_). TxA_2_ is a metabolite derived from AA via the COX pathway, a potent vasoconstrictor, and a platelet agonist that results in irreversible platelet aggregation [53,54,55]. The inhibitory potential of S-G04 was comparable to that of Indomethacin, a COX inhibitor (Figure 7F). The data in Figure 7 show the potential of the RhoA inhibitors to inhibit platelet aggregation mediated by diverse agonists, i.e., TxA2, ADP, U46619, AA, and thrombin, and ATP secretion, highlighting the potential to inhibit multiple-agonist-mediated platelet activation pathways at micromolar concentrations.

## 3. Materials and Methods

### 3.1. Experimental Section

Rhosin/RG04 was custom-synthesized as described [23]. Collagen (No. P/N 385), adenosine diphosphate (ADP) (No. P/N 384), adenosine triphosphate (ATP) standard (No. P/N 387), thrombin (No. P/N 386), and Chrono-lume^®^ (No. P/N 395) were obtained from Chronolog Corporation (Havertown, PA). Arachidonic acid (AA) (No. 90010) and U-46619 (No. 16450) were obtained from Chronolog Corporation (Havertown, PA). Chemicals and reagents were purchased from Sigma-Aldrich (St. Louis, MO, USA) or specifically noted sources. Anti-RhoA (67B9) antibody (No. 2117), Phospho-Myosin light chain 2 (Ser19) antibody (MLC) (No. 3671), Myosin light chain 2 (D18E2) antibody (No. 8505), and GAPDH (14C10) antibody (No. 2118) were purchased from Cell Signaling Technology (Danvers, MA, USA). Amersham ECL rabbit IgG, HRP-linked whole Ab (from donkey) (No. NA934) was ordered from Cytiva (Marlborough, MA, USA).

### 3.2. Virtual Screening

The UC/CCHMC compound library of over 360,000 chemicals was scanned for Rhosin analogs using similarity and substructure searches. These cheminformatics searches were performed in Pipeline Pilot (version 18.1.100.11; Dassault Systemes BioVia). Similarity searches were based on ECFP4, FCFP4, and FCFC4 fingerprints using Tanimoto similarity calculations. This library was subjected to a virtual screening of the GDP/GTP binding pocket and the GEF binding site of RhoA. Virtual screening was performed in the ICM-Pro software suite (version 3.8-7; Molsoft LLC) against RhoA structures PDB ID 1Xx86 [52] and 1ftn [56] obtained from Protein Data Bank (available online at www.rcsb.org (accessed on 22 August 2022)) [57], and later dockings included 5C4M [58]. Individual PDB files (PDBIDs 1 × 86, 1ftn) were read in ICM-Pro and stripped of crystallographic artifacts and waters. The docking sites were defined by means of visual inspection in Pymol and verified with the Pocket-Finder function in MolSoft ICM-Pro. The above SD files were read in ICM-Pro and converted into 3D structures (50 low-energy conformers), and H atoms and Gasteiger charges were added; then, the structures were docked at thoroughness of 30.

### 3.3. Ultra-High-Performance Liquid Chromatography Coupled with High-Resolution Mass Spectrometry Analysis (UHPLC-HRMS)

Compounds in DMSO solution (1 mM) were further dissolved into acetonitrile/water (1/1 *v*/*v*) to the appropriate concentration for mass spectrometry. Volumes of 10 µL of prepared solution of S-GO4 and R-GO4, and 5 µL of analogs UC-177618 and UC-177619 were injected into a Q ExactiveTM plus hybrid quadrupole-OrbitrapTM mass spectrometer interfaced with a Vanquish ultra-high-performance liquid chromatography (UHPLC) system (Thermo Scientific, Waltham, MA, USA). Chromatography separation was conducted using an Acquity BEH C18 UPLC column (2.1 × 100 mm; 1.7 µm) with a guard column (Waters). The column temperature was set to 20 °C. An isocratic mobile phase was used with a binary solvent system, and 65% solvent A (water with 2 mM ammonium acetate and 0.1% formic acid) and 35% solvent B (acetonitrile) were used in the analysis. The total run time was 10 min, and the flow rate was 0.2 mL/min. The ESI source was operated with the following parameters: spray voltage, 3.4 kV; capillary temperature, 350 °C; sheath gas flow rate, 35; auxiliary gas heater temperature, 325 °C. Data were acquired using a full MS scan (mass scan range of 150–900 *m*/*z*, AGC target of 3e6, maximum IT of 100 ms, and resolution of 70,000), and collision-induced dissociation-based data were dependent on MS/MS (resolution of 17,500, AGC target of 1e5, maximum IT of 50 ms, loop count of 7, top N = 7, isolation window of 1.0 *m*/*z*, and stepped NCE of 25). Samples were also checked with an additional gradient LC method to confirm the results. Data were analyzed with Xcalibur™ data acquisition and interpretation software and FreeStyle™ 1.6 (Thermo Scientific).

### 3.4. Collection of Blood and Preparation of Washed Human Platelet Suspensions

All experiments using human blood from healthy volunteers were performed according to the protocols approved by the Institutional Review Board at Cincinnati Children’s Hospital Medical Center, Cincinnati, Ohio. Each volunteer was required to sign an informed consent form approved by the IRB. Procedures for drawing human blood, isolating platelet-rich plasma (PRP), and preparing washed platelet suspensions were the same as those previously reported [59,60]. Blood from healthy human donors who reported having been free of medication for ten days was collected into ACD (2.5% trisodium citrate, 2% dextrose, and 1.5% citric acid) at 1:6 (*v*/*v*). PRP was obtained by means of the centrifugation of blood at 1400 rpm for 15 min at room temperature. PRP was centrifuged at 4000 rpm for 10 min at room temperature, and the platelet pellet was resuspended in HEPES Tyrode’s solution (pH 7.4) without calcium. Platelets were counted using Hemavet 950FS (Drew Scientific, Oxford, CT, USA), and the count was adjusted to 2.5 × 10^8^/mL for aggregation studies.

### 3.5. Lactate Dehydrogenase (LDH)-Based Cytotoxicity Assay

Compound cytotoxicity was assessed using LDH Cytotoxicity Assay Kit (No. 601170; Cayman Chemical, MI) as previously described [61]. Washed human platelets (2.5 × 10^8^/mL) were incubated at 37 °C for 10 min with the highest concentration of compounds (50 µM for R-G04 and S-G04, and 20 µM for Rhosin analogs) or the vehicle, dimethyl sulfoxide (DMSO) 0.1% or 10% Triton X-100, which served as the positive control. The platelets were centrifuged at 800× *g* for 8 min at 4 °C, and 100 µL of the supernatant was mixed with 100 µL of LDH reaction solution in a 96-well plate. The plate was incubated at 37 °C for 10 min under constant orbital shaking before measurement of the absorbance at 490 nm in a plate reader (BioTek Synergy LX multimode reader; Santa Clara, CA, USA).

### 3.6. Antiplatelet Aggregation Activity and Secretion of ATP

The in vitro antiplatelet activity of the compounds was assayed on washed human platelets using light transmission aggregometry [36]. Platelet aggregation was performed using a 4-channel Lumi-aggregometer (Chronolog, PA, USA) at 37 °C and stirring speed of 900 rpm [14]. A volume of 250 µL of washed human platelets (2.5 × 10^8^/mL) was pre-warmed to 37 °C along with the compounds or DMSO (0.1%) for 2 min before the addition of agonists such as collagen, AA, U46619 (a stable synthetic analog of thromboxane A_2_), ADP, and thrombin. For ATP secretion, platelets were pre-incubated with a luciferin/luciferase kit (Chrono-lume^®^) for 30 s at 37 °C before the addition of the agonist [60]. The reactions were allowed to proceed for at least 4 min, and the results of platelet aggregation and ATP secretion were determined using Aggrolink software. Platelet aggregation was expressed as the maximum change in the light transmission, where the 100% value corresponded to treatment with the agonist alone. Compounds were evaluated in triplicate at nine compound concentrations ranging from 50 µM to 1 µM. The half-maximal inhibitory concentration (IC_50_) value was calculated as the concentration of the inhibitor causing 50% inhibition of aggregation.

### 3.7. RhoA GTPase Assay and Phosphorylation of MLC

The relative levels of RhoA-GTP in washed human platelets were quantified with effector domains GST-Rhotekin pull-down assays as previously reported [23]. A volume of 250 µL of washed human platelets (2.5 × 10^8^/mL) was pre-warmed to 37 °C along with the compounds or DMSO (0.1%) for 2 min before being stimulated with collagen (1 µg/mL). The reactions were terminated 4 min after collagen stimulation by adding ice-cold lysis buffer (20 mM Tris·HCl (pH 7.6), 100 mM NaCl, 1% Triton X-100, 0.2% Sodium Deoxycholate, 10 mM MgCl2, 1 mM dithiothreitol, 1x protease inhibitor cocktail, and 1x phosphatase inhibitor cocktail). The supernatants were collected and subjected to the GST-Rhotekin pull-down assay. The total cell lysates were also blotted in parallel. GTP-bound RhoA was quantitatively detected with Western blotting using an anti-RhoA antibody. The relative amounts of RhoA were quantified with densitometry measurements and normalized to the untreated platelets.

A volume of 250 µL of washed human platelets (2.5 × 10^8^/mL) was pre-warmed to 37 °C along with the compounds or DMSO (0.1%) for 2 min before being stimulated with collagen (1 µg/mL) for 4 min. The reactions were terminated by adding 4x Laemmli buffer, and phosphorylated MLC protein was detected with Western blotting as previously reported [15]. Phosphorylation was quantified by measuring the densitometry.

### 3.8. In Vitro Complex-Formation Assay

In total, 100 ng of purified MBP (Maltose-binding protein)-(His)6-tagged LARG DH-PH was immobilized on 20 µL of suspended amylose beads (No. E8021S; New England Biolabs Inc., Ipswich, MA, USA) by incubating them in an orbital shaker at 4 °C for 30 min in binding buffer (20 mM Tris·HCl (pH 7.6), 100 mM NaCl, 0.01% Triton X-100, 5mM EDTA, and 5 mM MgCl_2_). The compounds were added to the binding buffer containing 100 ng of purified full-length RhoA and pre-incubated at 4 °C for 30 min under constant agitation. The Amylose beads bound to LARG were washed thrice with binding buffer to get rid of unbound LARG, and the pre-incubated compound-RhoA mixture was added to the beads. This LARG-compound-RhoA mixture was incubated in the orbital shaker at 4 °C for 1 h. The beads were washed three times, and the amount of RhoA protein co-precipitated with the MBP-fusion-bound beads was detected with anti-RhoA Western blotting.

### 3.9. Thromboxane B2 ELISA Assay

The level of TxB_2_ was measured with an enzyme immunoassay kit (No. 501020; Cayman Chemical, Ann Arbor, MI, USA) as previously described [32]. A volume of 250 µL of washed human platelets (2.5 × 10^8^/mL) was pre-warmed to 37 °C along with the Rhosin-analogs, Indomethacin, or DMSO (0.1%) for 2 min before being stimulated with thrombin (0.1 U/mL) for 4 min. The reactions were terminated by adding 250 µL of HEPES Tyrode’s buffer (pH 7.4) containing 2 mM EDTA. The platelets were centrifuged for 2 min, and the level of TxB_2_ in the supernatant was measured with the ELISA kit according to the manufacturer’s instructions.

### 3.10. Statistical Analysis

Data were obtained from experiments on samples from at least three healthy volunteers and expressed as means ± standard deviations (SDs) where applicable. All experimental data were analyzed using Prism 9.0 software/Graph Pad Inc., San Diego, CA, USA and compared for statistically significant differences using two-tailed Student’s *t*-test.

## 4. Conclusions and Future Directions

We evaluated G04 stereoisomers and a series of closely related compounds in a platelet activity screening to identify a new generation of small-molecule RhoA inhibitors. We determined that the S enantiomer of G04 has significantly higher potency than the initial lead Rhosin/R-G04, and we identified a series of analogs with even higher potency. Based on our SAR analysis, we conclude that an ideal RhoA inhibitor must have a quinoline attached to the hydrazine at the 4-position and that having a halogen substitution at the 7- or 8-position is beneficial. Comparable potency was seen in the indole and methylphenyl substituents, with slightly better potency having been observed in the dicholorphenyl group. The replacement of the quinoline moiety with an indane afforded a nearly equipotent compound, while a positional isomer lost all activity. The G04 stereoisomers and the active analogs exhibited a similar mechanism of inhibition of RhoA, whereby they inhibited the binding of LARG to RhoA in the complex-formation assay. Additional platelet activation assays indicated that this class of RhoA inhibitors possesses broad antiplatelet properties.

Although this work focused on testing the antiplatelet activity of these compounds, the lack of knowledge about their biodisponibility, pharmacokinetics, and pharmacodynamics is a limit to their pharmacological advancement. This limitation can be addressed using in vivo systems and animal models. We realize that the study lacks a deeper understanding of the mechanism of action of RhoA inhibitors; this could be addressed in the future using nuclear magnetic resonance or crystallography (NMR/crystallography) and additional biophysical and biochemical assays. Due to the reversible nature of these inhibitors and their ability to inhibit multiple-agonist-mediated platelet aggregation, they have the potential to be developed into broad and reversible antiplatelet alternative agents to the available antiplatelet drugs on the market, adding to the currently available indomethacin and cangrelor, which work according to distinct mechanisms. Further studies are necessary to understand the pharmacological profile of these RhoA inhibitors and to help them further advance into clinical trials.

## Figures and Tables

**Table 1:** Structures of Rhosin-related compounds and their effects at 20 µM concentration on in vitro collagen-induced platelet aggregation.

Derivative	Structure	% Platelet Aggregation ^b^	
		Collagen (1 µg/mL)	Collagen (5 µg/mL)
UC-177629	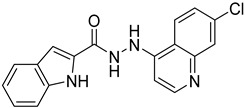	7.3 ± 5.9	10.8 ± 3
UC-391695	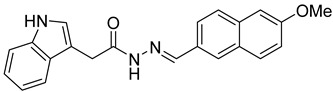	79 ± 8	84.8 ± 12.1
UC-436352	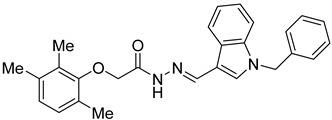	84.7 ± 15.5	89 ± 2.5
UC-392453	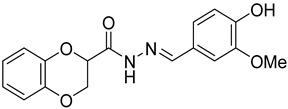	90.7 ± 8.5	94.7 ± 11.2
UC-830013	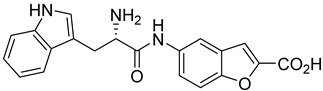	93 ± 10.8	91.3 ± 14.7
UC-984842	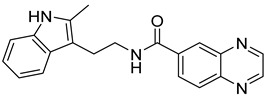	91.3 ± 10.5	95.9 ± 6.2
UC-651009	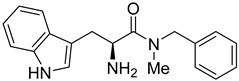	86 ± 1.7	89.2 ± 10.6
Indomethacin ^a^		12 ± 4.4	69 ± 12.3
Cangrelor ^a^		27.67 ± 2.52	63.33 ± 4.16

^a^, Indomethacin and Cangrelor (20 µM) were used as positive controls; ^b^, values are presented as means ± SDs of three independent experiments.

**Table 2:** Structures of compound UC-177629 analogs and their effects at 20 µM on in vitro collagen-induced platelet aggregation.

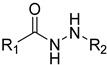				
**Derivative**	**R_1_**	**R_2_**	**% Platelet Aggregation ^b^**	
			**Collagen (1 µg/mL)**	**Collagen (5 µg/mL)**
UC-177626	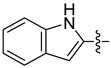	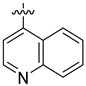	86 ± 14.4	87.6 ± 19.4
UC-177629	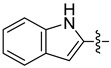	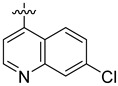	7.3 ± 5.9	10.8 ± 3
UC-177627	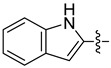	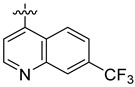	17.7 ± 11.4	19 ± 17.7
UC-177628	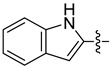	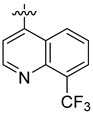	8.7 ± 4.7	12.3 ± 9
UC-177630	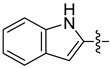	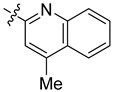	73 ± 21.6	85 ± 15.3
UC-178838	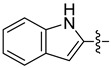	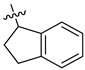	7.3 ± 5.8	13.7 ± 7.9
UC-177617	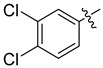	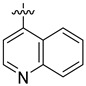	13 ± 10.4	26.8 ± 9
UC-177619	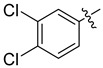	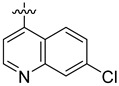	9.3 ± 3.5	12 ± 2.1
UC-177618	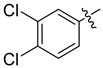	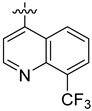	5 ± 1.7	10.2 ± 4.1
UC-177631	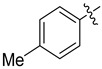	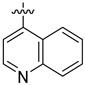	87.7 ± 13.1	90.4 ± 3.7
UC-177634	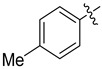	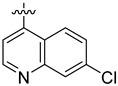	17 ± 2.6	24.5 ± 3.9
UC-177632	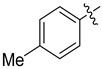	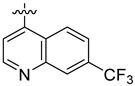	86.7 ± 11	93.1 ± 12.8
UC-177633	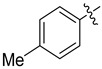	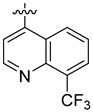	3.3 ± 3.2	10.1 ± 8.3
UC-177635	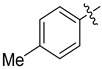	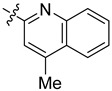	79.1 ± 7	90.1 ± 8.3
UC-177639	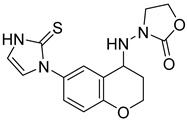		80 ± 17.3	90.4 ± 9.1
UC-390484	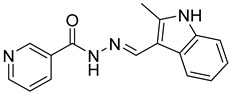		81 ± 14	88.1 ± 10
UC-406794	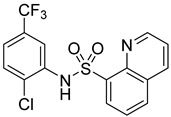		12.7 ± 7.2	25 ± 5.2
UC-612333	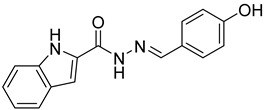		70 ± 5.6	78.6 ± 14.9
UC-936377	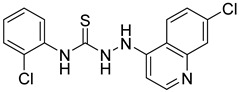		61.3 ± 4.5	71.7 ± 5.3
UC177623	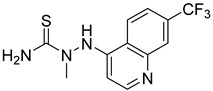		84.3 ± 9	90.7 ± 6.2
UC-177613	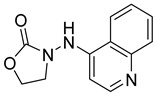		84.3 ± 15.5	95.2 ± 4.9
UC-177614	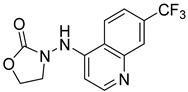		87.3 ± 9.6	90 ± 3.1
UC-177615	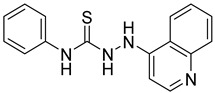		9.7 ± 6	11.4 ± 9.9
UC-177616	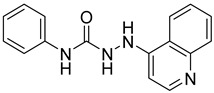		11.7 ± 2.3	10.6 ± 3.6
Indomethacin ^a^			12 ± 4.4	69 ± 12.3
Cangrelor ^a^			27.67 ± 2.52	63.33 ± 4.16

^a^, Indomethacin and Cangrelor (20 µM) were used as positive controls; ^b^, values are presented as means ± SDs of three independent experiments.

**Table 3:** Structure–activity relationship of UC-177629 analogs derived by arranging the compounds with variations in the quinoline group across the vertical axis and placing compounds with variations in their aryl group across the horizontal axis.

	**Aryl Group**	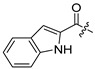	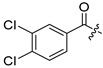	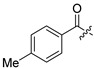
**Quinoline Group**				
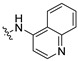		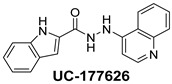 IC_50_ > 20 µM	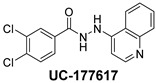 IC_50_ = 5.64 µM	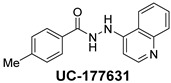 IC_50_ > 20 µM
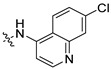		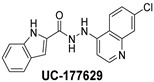 IC_50_ = 2.39 µM	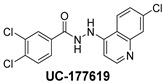 IC_50_ = 0.82 µM	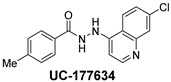 IC_50_ = 4.59 µM
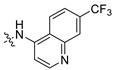		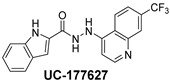 IC_50_ = 2.23 µM		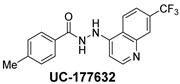 IC_50_ > 20 µM
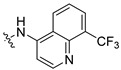		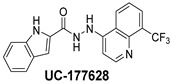 IC_50_ = 2.84 µM	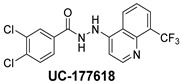 IC_50_ = 1.5 µM	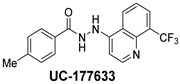 IC_50_ = 2.34 µM
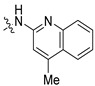		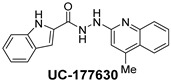 IC_50_ > 20 µM		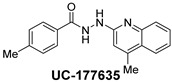 IC_50_ > 20 µM
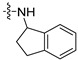		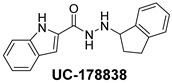 IC_50_ = 3.55 µM		

**Table 4:** Structures of Rhosin/R-G04 and its S-enantiomer, S-G04, and their effects at 20 µM on in vitro collagen-induced platelet aggregation.

Derivative	Structure	% Platelet Aggregation ^b^	
		Collagen (1 µg/mL)	Collagen (5 µg/mL)
Rhosin/ R-G04	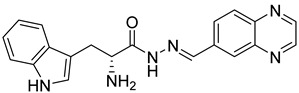	70.33 ± 18.90	71.36 ± 15.01
S-G04	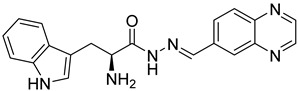	9.33 ± 4.93	20 ± 5
Indomethacin ^a^		12 ± 4.4	69 ± 12.3
Cangrelor ^a^		27.67 ± 2.52	63.33 ± 4.16

^a^, Indomethacin and Cangrelor (20 µM) were used as positive controls; ^b^, values are presented as means ± SDs of three independent experiments.

## Data Availability

Data available within the article or its Supplementary Materials.

## Supplementary Materials

The following supporting information can be downloaded at: https://www.mdpi.com/article/10.3390/ijms24044167/s1.

## References

[1] Versteeg H.H., Heemskerk J.W., Levi M., Reitsma P.H. (2013). New fundamentals in hemostasis. Physiol. Rev..

[2] Kakouros N., Rade J.J., Kourliouros A., Resar J.R. (2011). Platelet function in patients with diabetes mellitus: From a theoretical to a practical perspective. Int. J. Endocrinol..

[3] Lievens D., von Hundelshausen P. (2011). Platelets in atherosclerosis. Thromb. Haemost..

[4] Lip G.Y.H. (2003). Hypertension, Platelets, and the Endothelium. Hypertension.

[5] Fuster V., Sweeny J.M. (2011). Aspirin. Circulation.

[6] Degrauwe S., Pilgrim T., Aminian A., Noble S., Meier P., Iglesias J.F. (2017). Dual antiplatelet therapy for secondary prevention of coronary artery disease. Open Heart.

[7] Eikelboom J.W., Hirsh J., Spencer F.A., Baglin T.P., Weitz J.I. (2012). Antiplatelet drugs: Antithrombotic Therapy and Prevention of Thrombosis, 9th ed: American College of Chest Physicians Evidence-Based Clinical Practice Guidelines. Chest.

[8] Iqbal A.M., Lopez R.A., Hai O. (2022). Antiplatelet Medications.

[9] Thachil J. (2016). Antiplatelet therapy—A summary for the general physicians. Clin. Med..

[10] Angiolillo D.J. (2009). Variability in responsiveness to oral antiplatelet therapy. Am. J. Cardiol..

[11] Di Minno M.N., Guida A., Camera M., Colli S., Di Minno G., Tremoli E. (2011). Overcoming limitations of current antiplatelet drugs: A concerted effort for more profitable strategies of intervention. Ann. Med..

[12] Shen B., Delaney M.K., Du X. (2012). Inside-out, outside-in, and inside-outside-in: G protein signaling in integrin-mediated cell adhesion, spreading, and retraction. Curr. Opin. Cell. Biol..

[13] Flevaris P., Li Z., Zhang G., Zheng Y., Liu J., Du X. (2009). Two distinct roles of mitogen-activated protein kinases in platelets and a novel Rac1-MAPK-dependent integrin outside-in retractile signaling pathway. Blood.

[14] Akbar H., Shang X., Perveen R., Berryman M., Funk K., Johnson J.F., Tandon N.N., Zheng Y. (2011). Gene targeting implicates Cdc42 GTPase in GPVI and non-GPVI mediated platelet filopodia formation, secretion and aggregation. PLoS ONE.

[15] Akbar H., Kim J., Funk K., Cancelas J.A., Shang X., Chen L., Johnson J.F., Williams D.A., Zheng Y. (2007). Genetic and pharmacologic evidence that Rac1 GTPase is involved in regulation of platelet secretion and aggregation. J. Thromb. Haemost..

[16] Pandey D., Goyal P., Dwivedi S., Siess W. (2009). Unraveling a novel Rac1-mediated signaling pathway that regulates cofilin dephosphorylation and secretion in thrombin-stimulated platelets. Blood.

[17] Dwivedi S., Pandey D., Khandoga A.L., Brandl R., Siess W. (2010). Rac1-mediated signaling plays a central role in secretion-dependent platelet aggregation in human blood stimulated by atherosclerotic plaque. J. Transl. Med..

[18] Akbar H., Cancelas J., Williams D.A., Zheng J., Zheng Y. (2006). Rational design and applications of a Rac GTPase-specific small molecule inhibitor. Methods Enzymol..

[19] Klages B., Brandt U., Simon M.I., Schultz G., Offermanns S. (1999). Activation of G12/G13 results in shape change and Rho/Rho-kinase-mediated myosin light chain phosphorylation in mouse platelets. J. Cell. Biol..

[20] Pleines I., Hagedorn I., Gupta S., May F., Chakarova L., van Hengel J., Offermanns S., Krohne G., Kleinschnitz C., Brakebusch C. (2012). Megakaryocyte-specific RhoA deficiency causes macrothrombocytopenia and defective platelet activation in hemostasis and thrombosis. Blood.

[21] Offermanns S. (2006). Activation of platelet function through G protein-coupled receptors. Circ. Res..

[22] Kim J.S., Kim J.G., Jeon C.Y., Won H.Y., Moon M.Y., Seo J.Y., Kim J.I., Kim J., Lee J.Y., Choi S.Y. (2005). Downstream components of RhoA required for signal pathway of superoxide formation during phagocytosis of serum opsonized zymosans in macrophages. Exp. Mol. Med..

[23] Shang X., Marchioni F., Sipes N., Evelyn C.R., Jerabek-Willemsen M., Duhr S., Seibel W., Wortman M., Zheng Y. (2012). Rational design of small molecule inhibitors targeting RhoA subfamily Rho GTPases. Chem. Biol..

[24] Shang X., Zheng Y. (2012). Rational design of Rho GTPase-targeting inhibitors. Methods Mol. Biol..

[25] Akbar H., Duan X., Saleem S., Davis A.K., Zheng Y. (2016). RhoA and Rac1 GTPases Differentially Regulate Agonist-Receptor Mediated Reactive Oxygen Species Generation in Platelets. PLoS ONE.

[26] Brito F.C., Kummerle A.E., Lugnier C., Fraga C.A., Barreiro E.J., Miranda A.L. (2010). Novel thienylacylhydrazone derivatives inhibit platelet aggregation through cyclic nucleotides modulation and thromboxane A2 synthesis inhibition. Eur. J. Pharmacol..

[27] Chelucci R.C., Dutra L.A., Lopes Pires M.E., de Melo T.R., Bosquesi P.L., Chung M.C., Dos Santos J.L. (2014). Antiplatelet and antithrombotic activities of non-steroidal anti-inflammatory drugs containing an N-acyl hydrazone subunit. Molecules.

[28] Haj Mohammad Ebrahim Tehrani K., Sardari S., Mashayekhi V., Esfahani Zadeh M., Azerang P., Kobarfard F. (2013). One Pot Synthesis and Biological Activity Evaluation of Novel Schiff Bases Derived from 2-Hydrazinyl-1,3,4-thiadiazole. Chem. Pharm. Bull..

[29] Klawans H.L., Ringel S.P., Shenker D.M. (1971). Failure of vitamin B6 to reverse the L-dopa effect in patients on a dopa decarboxylase inhibitor. J. Neurol. Neurosurg. Psychiatry.

[30] Mashayekhi V., Haj Mohammad Ebrahim Tehrani K., Amidi S., Kobarfard F. (2013). Synthesis of Novel Indole Hydrazone Derivatives and Evaluation of Their Antiplatelet Aggregation Activity. Chem. Pharm. Bull..

[31] McBride B.C., Van der Hoeven J.S. (1981). Role of interbacterial adherence in colonization of the oral cavities of gnotobiotic rats infected with Streptococcus mutans and Veillonella alcalescens. Infect. Immun..

[32] Park M.K., Rhee Y.H., Lee H.J., Lee E.O., Kim K.H., Park M.J., Jeon B.H., Shim B.S., Jung C.H., Choi S.H. (2008). Antiplatelet and antithrombotic activity of indole-3-carbinol in vitro and in vivo. Phytother. Res..

[33] Tavili N., Mokhtari S., Salehabadi H., Esfahanizadeh M., Mohebbi S. (2022). Novel N-substituted indole hydrazones as potential antiplatelet agents: Synthesis, biological evaluations, and molecular docking studies. Res. Pharm. Sci..

[34] Sterling T., Irwin J.J. (2015). ZINC 15—Ligand Discovery for Everyone. J. Chem. Inf. Model..

[35] PubChem National Library of Medicine (US), National Center for Biotechnology Information (2004). PubChem Compound Summary for CID 9552914, Rhosin. https://pubchem.ncbi.nlm.nih.gov/compound/Rhosin.

[36] Born G. (1962). Aggregation of Blood Platelets by Adenosine Diphosphate and Its Reversal.

[37] Baumgartner H.R., Haudenschild C. (1972). Adhesion of platelets to subendothelium. Ann. N. Y. Acad. Sci..

[38] Morton L.F., Peachey A.R., Barnes M.J. (1989). Platelet-reactive sites in collagens type I and type III. Evidence for separate adhesion and aggregatory sites. Biochem. J..

[39] Poole A.W., Watson S.P. (1995). Regulation of cytosolic calcium by collagen in single human platelets. Br. J. Pharmacol..

[40] Taylor M.L., Misso N.L., Stewart G.A., Thompson P.J. (1992). The effects of varying doses of aspirin on human platelet activation induced by PAF, collagen and arachidonic acid. Br. J. Clin. Pharmacol..

[41] Skovronsky D.M., Lee V.M., Pratico D. (2001). Amyloid precursor protein and amyloid beta peptide in human platelets. Role of cyclooxygenase and protein kinase C. J. Biol. Chem..

[42] Angiolillo D.J., Schneider D.J., Bhatt D.L., French W.J., Price M.J., Saucedo J.F., Shaburishvili T., Huber K., Prats J., Liu T. (2012). Pharmacodynamic effects of cangrelor and clopidogrel: The platelet function substudy from the cangrelor versus standard therapy to achieve optimal management of platelet inhibition (CHAMPION) trials. J. Thromb. Thrombolysis.

[43] Crook D., Collins A.J. (1977). Comparison of effects of aspirin and indomethacin on human platelet prostaglandin synthetase. Ann Rheum. Dis..

[44] Ferreiro J.L., Ueno M., Angiolillo D.J. (2009). Cangrelor: A review on its mechanism of action and clinical development. Expert. Rev. Cardiovasc. Ther..

[45] Hule V. (1966). Isoenzymes of lactic dehydrogenase in human platelets. Clin. Chim. Acta.

[46] Ravishankar D., Salamah M., Akimbaev A., Williams H.F., Albadawi D.A.I., Vaiyapuri R., Greco F., Osborn H.M.I., Vaiyapuri S. (2018). Impact of specific functional groups in flavonoids on the modulation of platelet activation. Sci. Rep..

[47] Cunha A.C., Figueiredo J.M., Tributino J.L., Miranda A.L., Castro H.C., Zingali R.B., Fraga C.A., de Souza M.C., Ferreira V.F., Barreiro E.J. (2003). Antiplatelet properties of novel N-substituted-phenyl-1,2,3-triazole-4-acylhydrazone derivatives. Bioorg. Med. Chem..

[48] Fraga A.G.M., Rodrigues C.R., de Miranda A.L.P., Barreiro E.J., Fraga C.A.M. (2000). Synthesis and pharmacological evaluation of novel heterotricyclic acylhydrazone derivatives, designed as PAF antagonists. Eur. J. Pharm. Sci..

[49] Khalid W., Badshah A., Khan A.U., Nadeem H., Ahmed S. (2018). Synthesis, characterization, molecular docking evaluation, antiplatelet and anticoagulant actions of 1,2,4 triazole hydrazone and sulphonamide novel derivatives. Chem. Cent. J..

[50] Lima L.M., Frattani F.S., Dos Santos J.L., Castro H.C., Fraga C.A., Zingali R.B., Barreiro E.J. (2008). Synthesis and anti-platelet activity of novel arylsulfonate--acylhydrazone derivatives, designed as antithrombotic candidates. Eur. J. Med. Chem..

[51] Faghih Akhlaghi M., Amidi S., Esfahanizadeh M., Daeihamed M., Kobarfard F. (2014). Synthesis of N-arylmethyl Substituted Indole Derivatives as New Antiplatelet Aggregation Agents. Iran. J. Pharm. Res..

[52] Kristelly R., Gao G., Tesmer J.J. (2004). Structural determinants of RhoA binding and nucleotide exchange in leukemia-associated Rho guanine-nucleotide exchange factor. J. Biol. Chem..

[53] Catella F., Healy D., Lawson J.A., FitzGerald G.A. (1986). 11-Dehydrothromboxane B_2_: A Quantitative Index of Thromboxane A<sub>2</sub> Formation in the Human Circulation. Proc. Natl. Acad. Sci. USA.

[54] FitzGerald G.A., Healy C., Daugherty J. (1987). Thromboxane A2 biosynthesis in human disease. Fed. Proc..

[55] Hamberg M., Svensson J., Samuelsson B. (1975). Thromboxanes: A new group of biologically active compounds derived from prostaglandin endoperoxides. Proc. Natl. Acad. Sci. USA.

[56] Wei Y., Zhang Y., Derewenda U., Liu X., Minor W., Nakamoto R.K., Somlyo A.V., Somlyo A.P., Derewenda Z.S. (1997). Crystal structure of RhoA-GDP and its functional implications. Nat. Struct. Biol..

[57] Berman H.M., Westbrook J., Feng Z., Gilliland G., Bhat T.N., Weissig H., Shindyalov I.N., Bourne P.E. (2000). The Protein Data Bank. Nucleic Acids Res..

[58] Pellegrini E., Bowleri M.W. (2016). RhoA GDP with novel switch II conformation. Protein Data Bank..

[59] Akbar H., Ardlie N.G. (1976). Evidence that collagen releases human platelet constituents by two different mechanisms. Br. J. Hematol..

[60] Akbar H., Duan X., Piatt R., Saleem S., Davis A.K., Tandon N.N., Bergmeier W., Zheng Y. (2018). Small molecule targeting the Rac1-NOX2 interaction prevents collagen-related peptide and thrombin-induced reactive oxygen species generation and platelet activation. J. Thromb. Haemost..

[61] Mendez D., Urra F.A., Millas-Vargas J.P., Alarcon M., Rodriguez-Lavado J., Palomo I., Trostchansky A., Araya-Maturana R., Fuentes E. (2020). Synthesis of antiplatelet ortho-carbonyl hydroquinones with differential action on platelet aggregation stimulated by collagen or TRAP-6. Eur. J. Med. Chem..

