# Supplementary material for: Structure–Activity Relationship Analysis of Rhosin, a RhoA GTPase Inhibitor, Reveals a New Class of Antiplatelet Agents
Source: Int J Mol Sci. 2023 Feb 19;24(4):4167. doi: 10.3390/ijms24044167 (PMC9961652; doi:10.3390/ijms24044167)
Supplement: Supplementary file 1 [file ijms-24-04167-s001.zip › ijms-2206142-supplementary.pdf]

A. B.

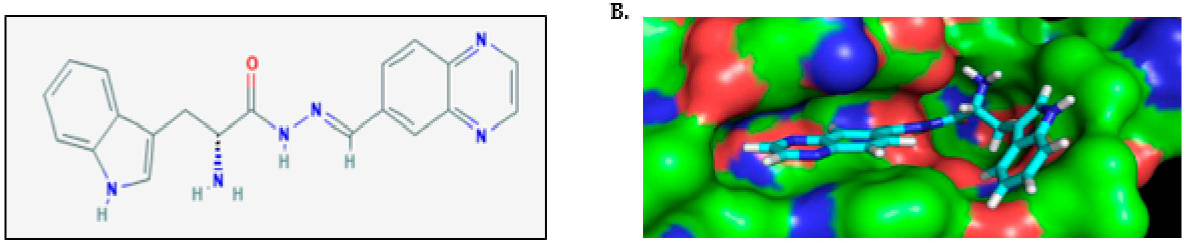

Figure S1. Rhosin binds to RhoA at the GEF site. (A) The chemical structure of Rhosin. (B) A simulated docking model of Rhosin on the surface of RhoA.

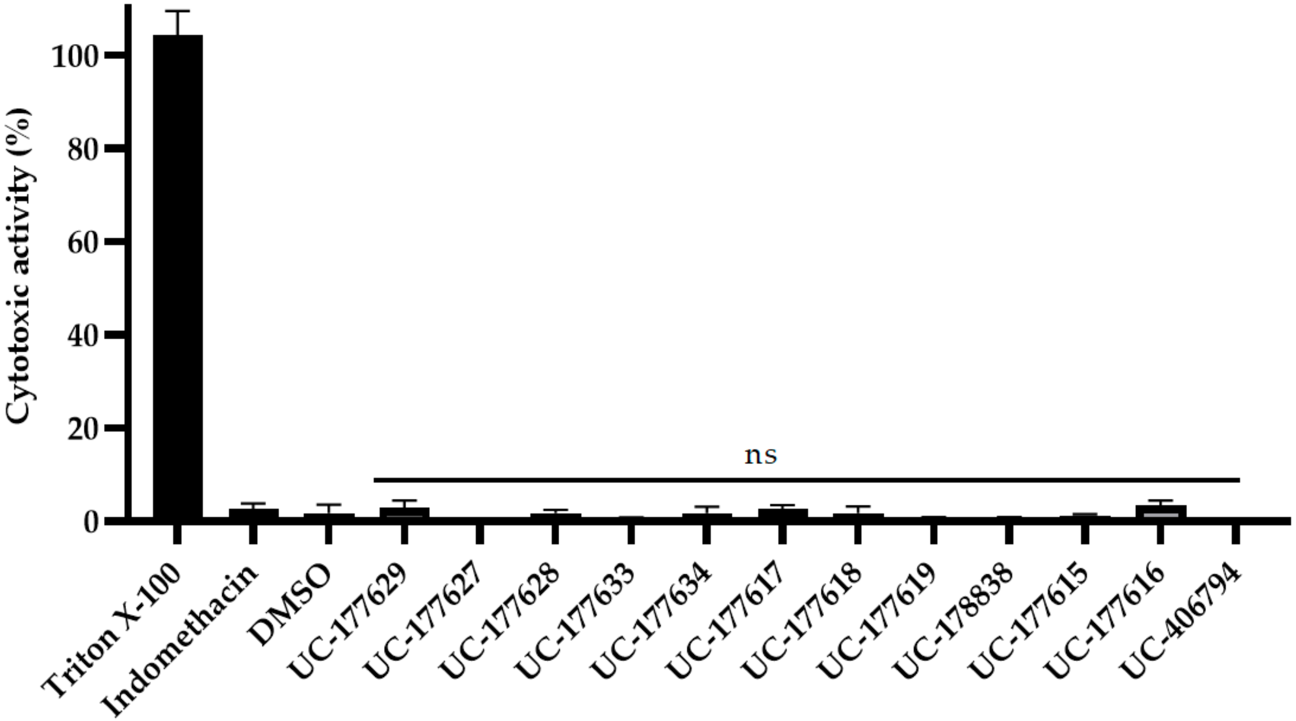

Figure S2. Cytotoxic activity of Rhosin-related compounds on washed human platelets determined by Lactate Dehydrogenase (LDH) release. Washed human platelets were incubated with either the Rhosin-related compounds, DMSO or Triton X-100, and the supernatants were used to measure the level of LDH release. The differences between the DMSO and the Rhosin-related compounds were analyzed by t-test, and the results are shown as mean±SD from three independent experiments.

A. UC-177618

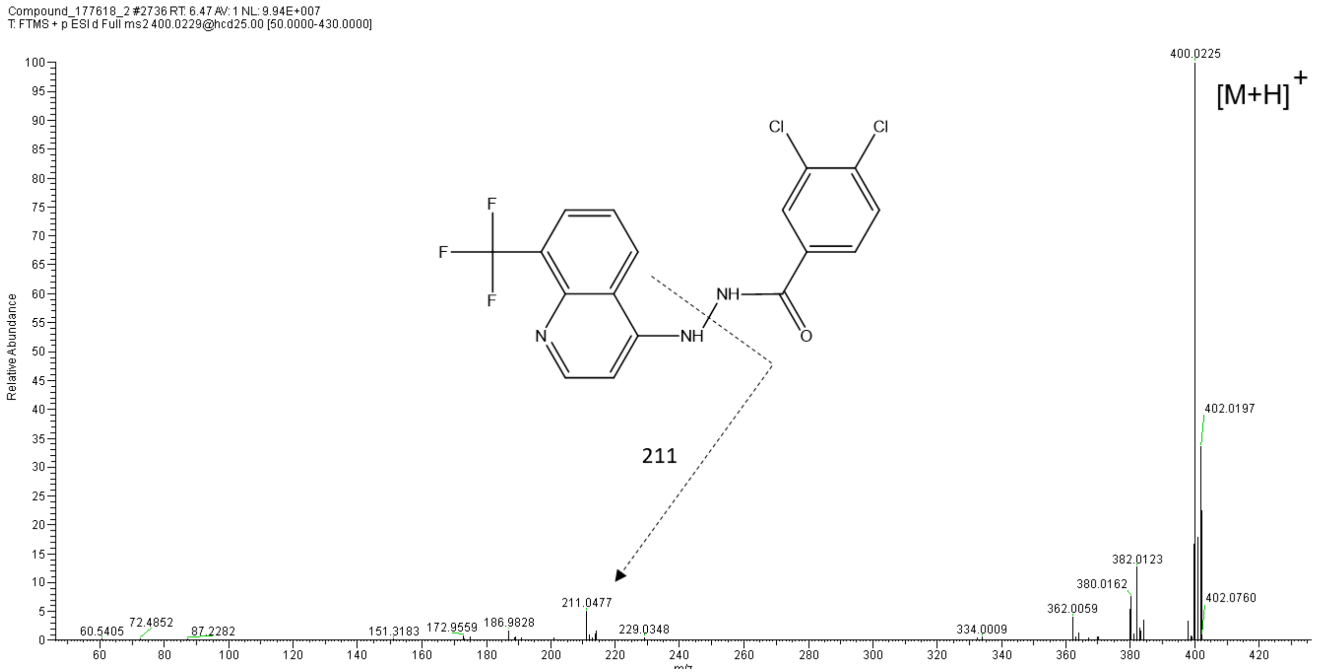

Compound_177618_2 #2736 RT: 6.47 AV: 1 NL: 9.94E+007
T: FTMS + p ESI d Full ms2 400.0229@hcd25.00 [50.0000-430.0000]
400.0225
[M+H]+
402.0197
382.0123
380.0162
362.0059
402.0760
211.0477
211
229.0348
334.0009
186.9828
172.9559
151.3183
87.2282
72.4852
60.5405
Relative Abundance
Cl
Cl
F
F
F
N
NH
NH
O

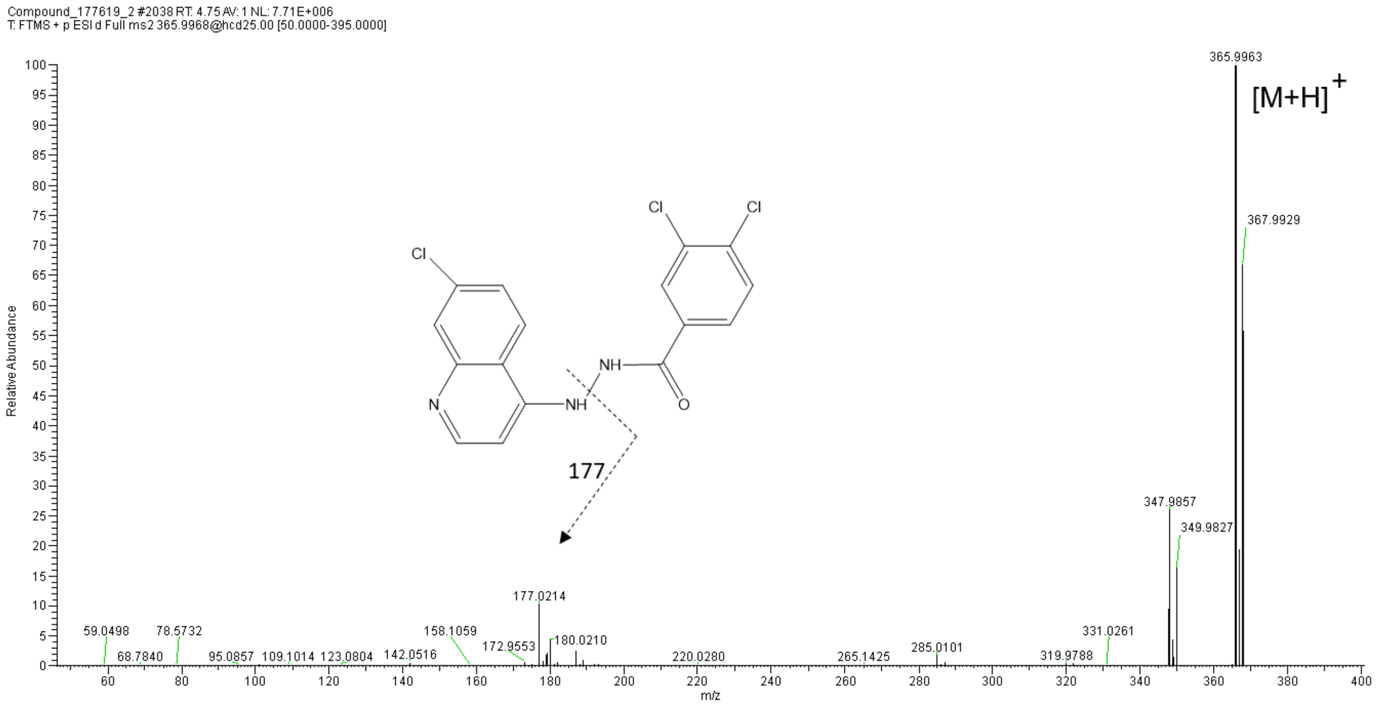
Compound_177619_2 #2038 RT: 4.75 AV: 1 NL: 7.71E+006
T: FTMS + p ESI d Full ms2 365.9968@hcd25.00 [50.0000-395.0000]
365.9963
[M+H]+
367.9929
347.9857
349.9827
177.0214
180.0210
177
Relative Abundance
m/z
Cl
NH
O
N

B. UC-177619

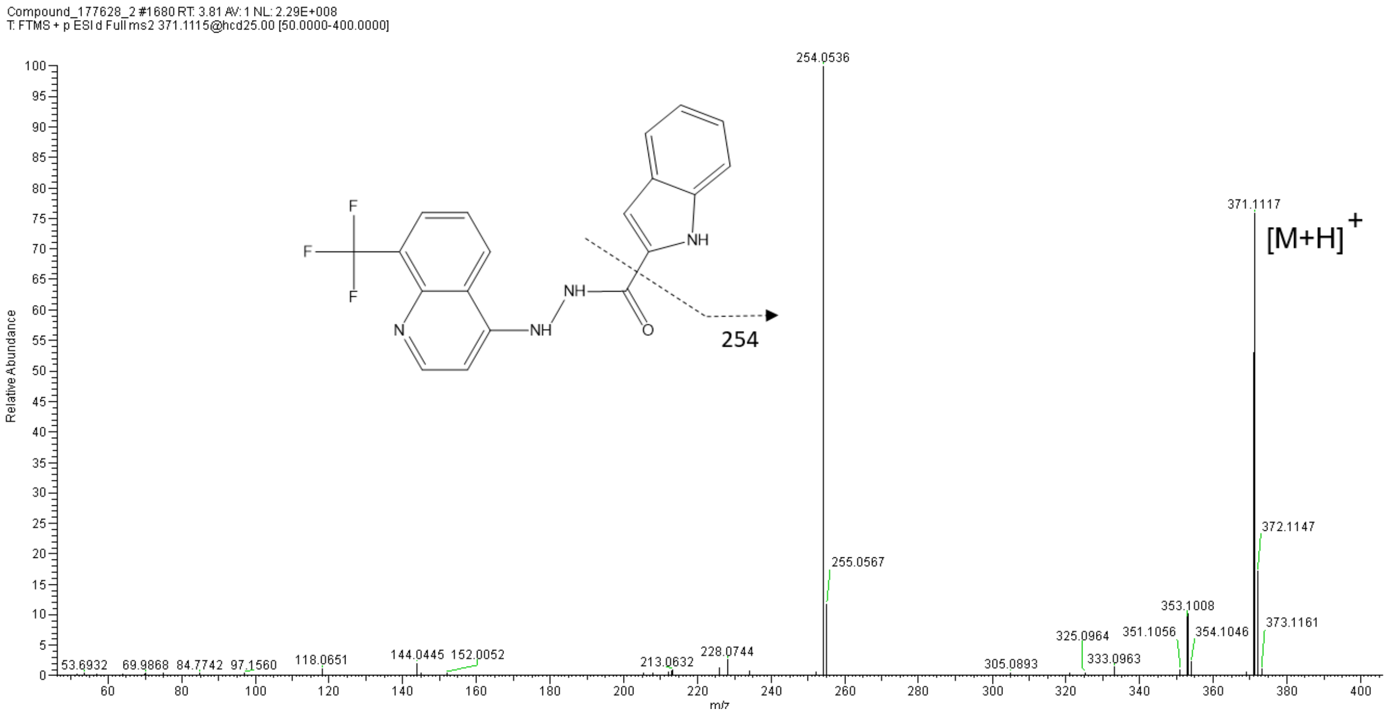

Compound_177628_2 #1680 RT: 3.81 AV: 1 NL: 2.29E+008
T: FTMS + p ESI d Full ms2 371.1115@hcd25.00 [50.0000-400.0000]
254.0536
371.1117
[M+H]+
254
372.1147
255.0567
353.1008
325.0964
351.1056
354.1046
373.1161
333.0963
305.0893
53.6932
69.9868
84.7742
97.1560
118.0651
144.0445
152.0052
213.0632
228.0744
Relative Abundance
m/z

C. UC-177628

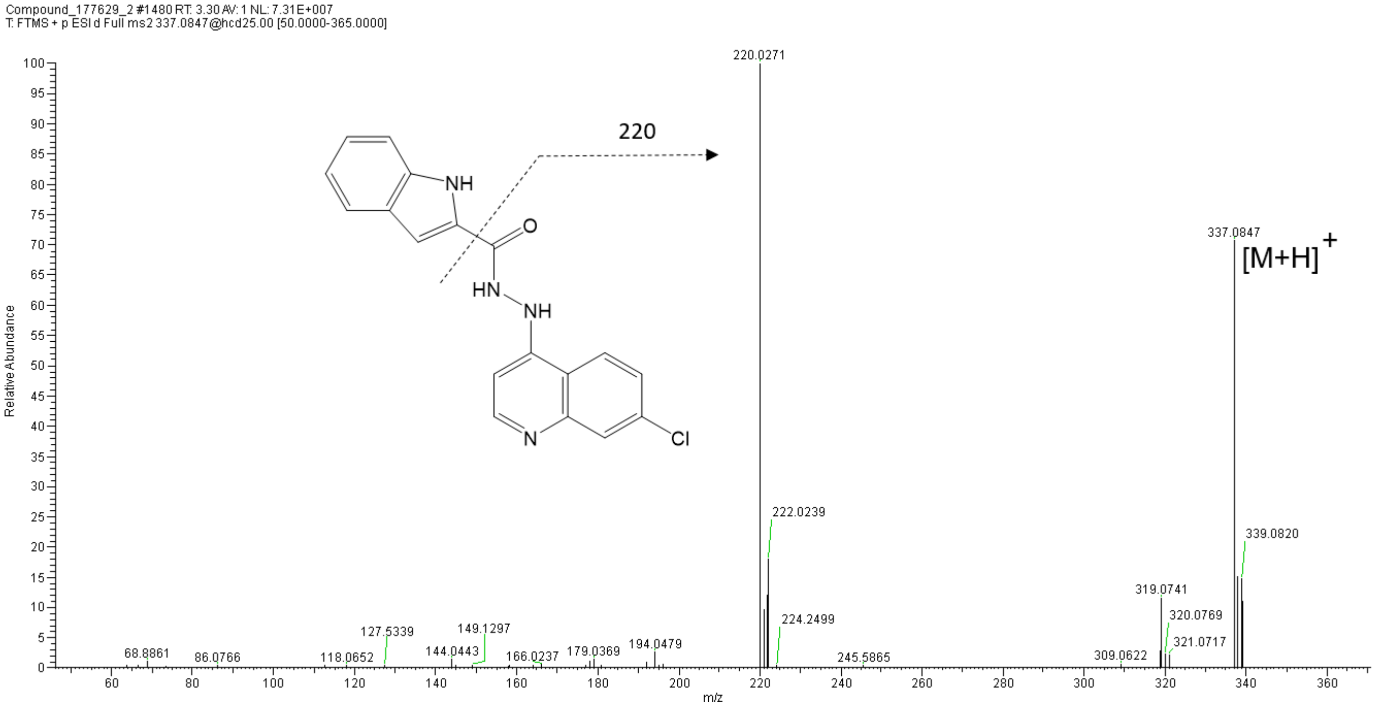

Compound_177629_2 #1480 RT: 3.30 AV: 1 NL: 7.31E+007
T: FTMS + p ESI d Full ms2 337.0847@hcd25.00 [50.0000-365.0000]
220
NH
O
HN
NH
N
Cl
220.0271
337.0847
[M+H]+
222.0239
339.0820
319.0741
320.0769
321.0717
224.2499
309.0622
245.5865
194.0479
179.0369
166.0237
149.1297
144.0443
127.5339
118.0652
86.0766
68.8861
Relative Abundance
m/z

D. UC-177629

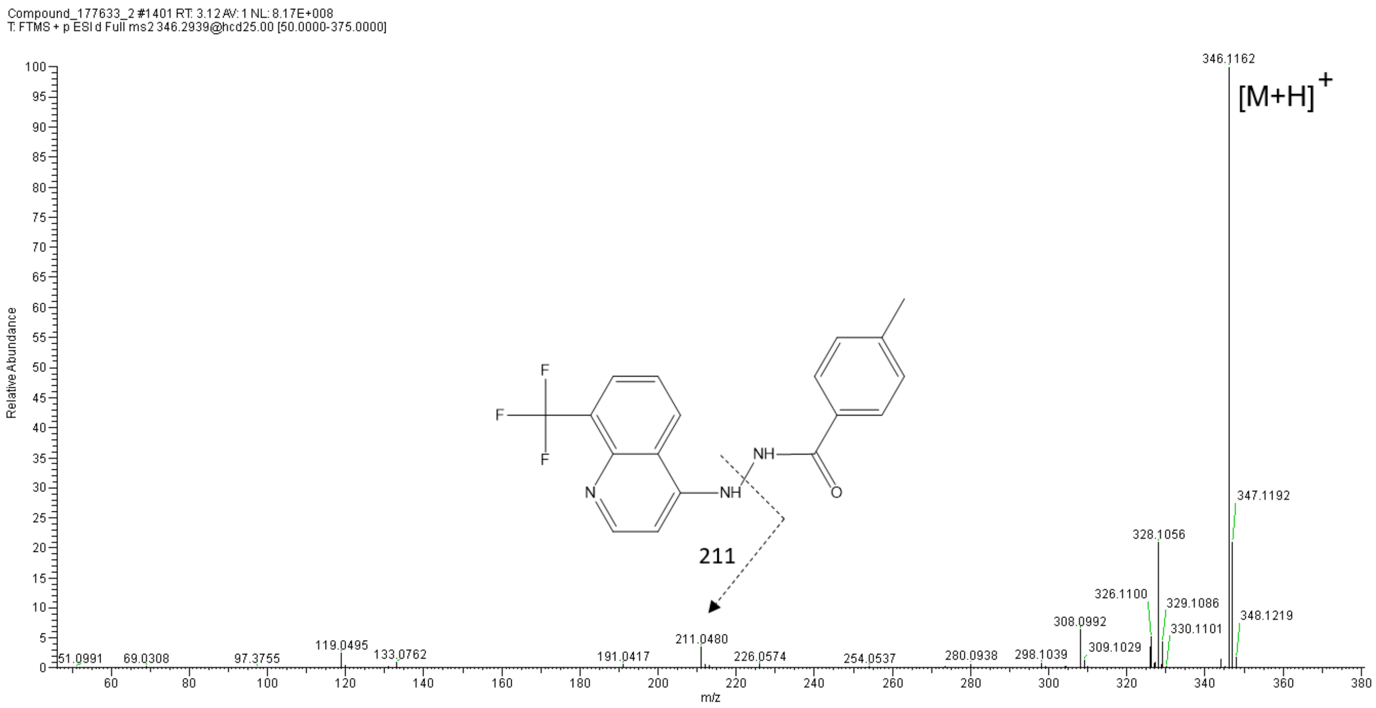
Compound_177633_2 #1401 RT: 3.12 AV: 1 NL: 8.17E+008
T: FTMS + p ESI d Full ms2 346.2939@hcd25.00 [50.0000-375.0000]
346.1162
[M+H]+
347.1192
328.1056
326.1100
329.1086
330.1101
348.1219
308.0992
309.1029
298.1039
280.0938
254.0537
226.0574
211.0480
191.0417
133.0762
119.0495
97.3755
69.0308
51.0991
211
Relative Abundance
m/z

E. UC-177633

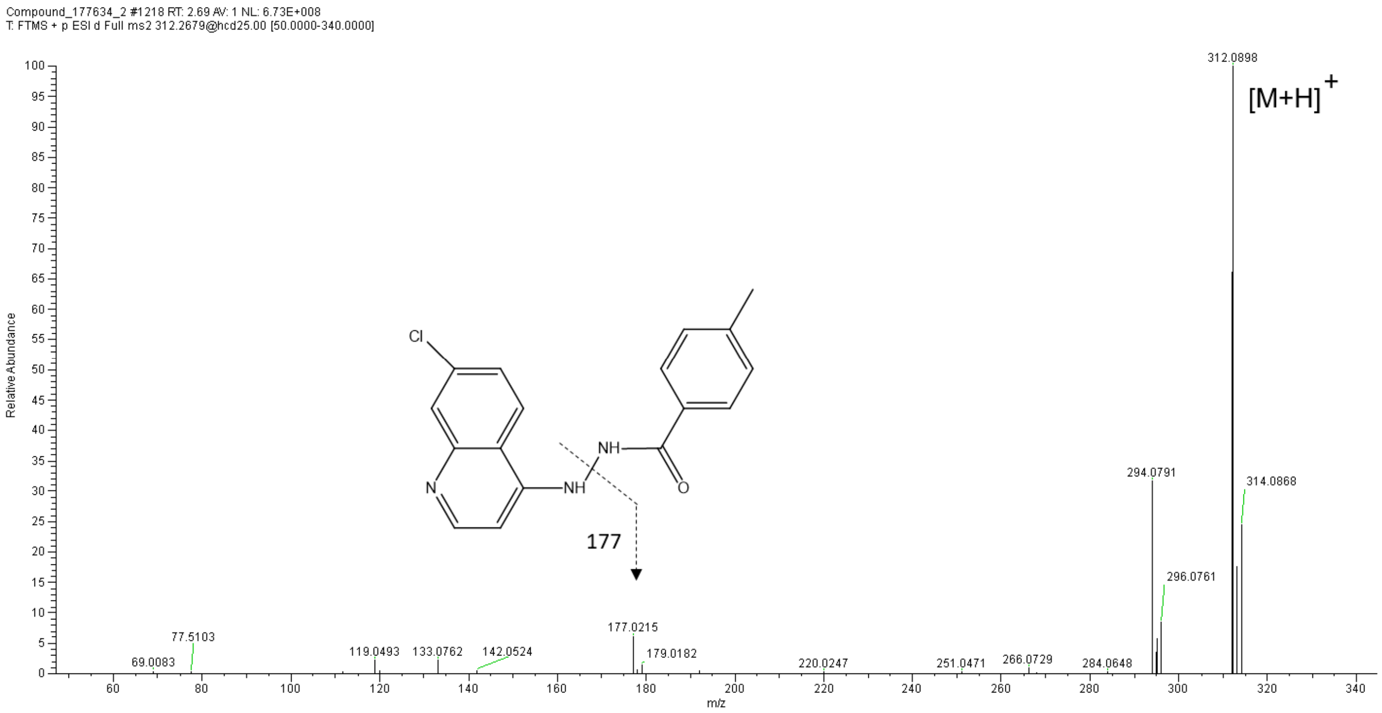

F. UC-177634

Figure S3. Fragmentation pattern peak to confirm the six active analogs. (A-F) UC-177618, UC-177619, UC-177628, UC-177629, UC-177633, and UC-177634 in DMSO solution were dissolved into acetonitrile/water (1/1 v/v). 5 µL of the prepared solution was injected into Q ExactiveTM plus hybrid quadrupole-OrbitrapTM mass spectrometer interfaced with Vanquish ultra-high performance liquid chromatography coupled with high-resolution mass spectrometry analysis (UHPLC-HRMS) (M represents the desired compound).

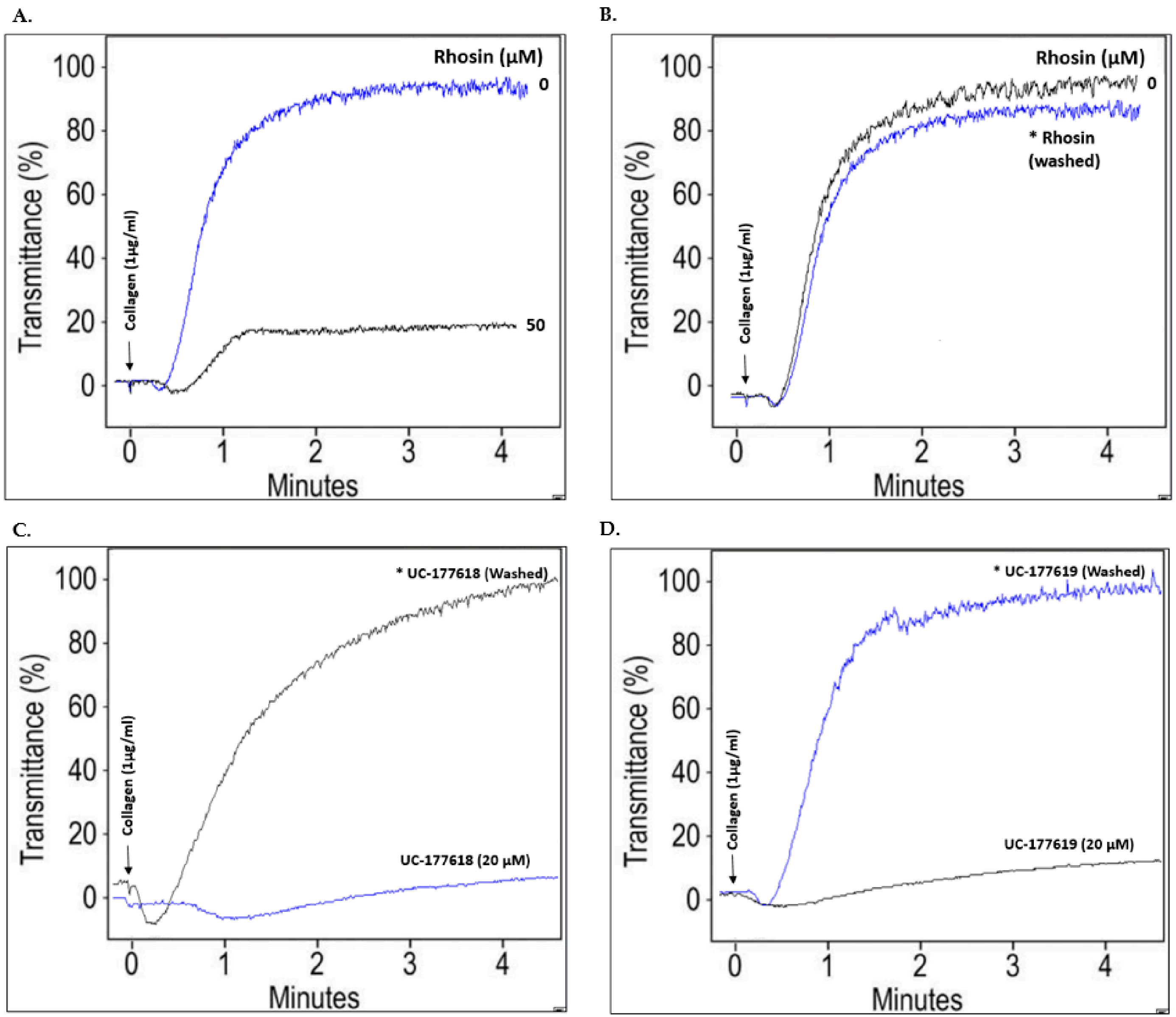

Figure S4. Rhosin and its active analogs, UC-177618 and UC-177619 are reversible inhibitors of collagen-induced platelet aggregation. (A) Pre-incubating washed human platelets with R-G04 (50 μM) for two minutes before stimulating with collagen (1 μg/ml) blocked aggregation. (B) However, human platelets incubated with R-G04 (50 μM) for 30 minutes at 37∘C and washed to remove the compound before stimulating with collagen exhibited normal aggregation. (C-D) Pre-incubating washed human platelets with UC-177618 (20 μM) or UC-177619 (20 μM) for two minutes before stimulating with collagen (1 μg/ml) blocked aggregation. However, human platelets incubated with UC-177618 (20 μM) or UC-177619 (20 μM) for

30 minutes at 37◦C and washed to remove the compound before stimulating with collagen exhibited normal aggregation. The aggregation tracings are representative of three independent experiments.

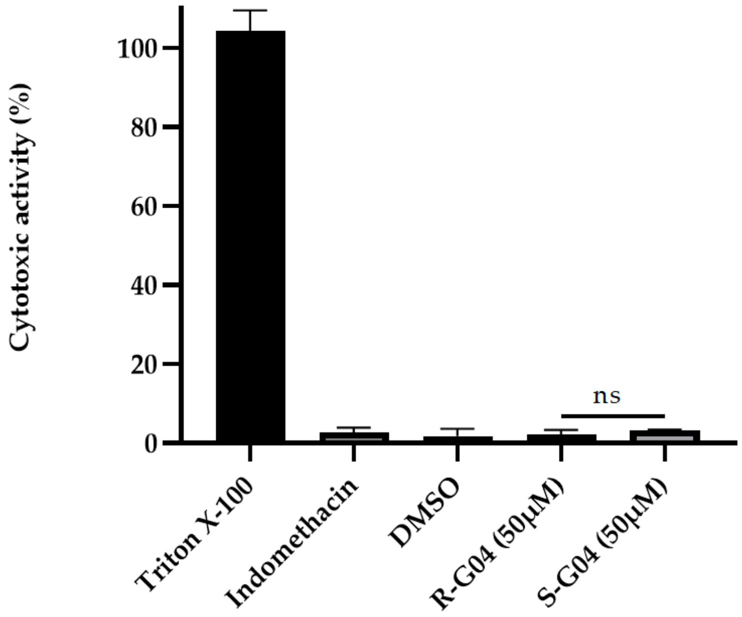

Figure S5. Cytotoxic activity of R-G04 and S-G04. Washed human platelets were incubated with R-G04, S-G04, DMSO, or Triton X-100, and the supernatants were used to measure the level of LDH release. The differences between the DMSO, R-G04, and S-G04 were analyzed by t-test, and the results are shown as mean±SD from three independent experiments.

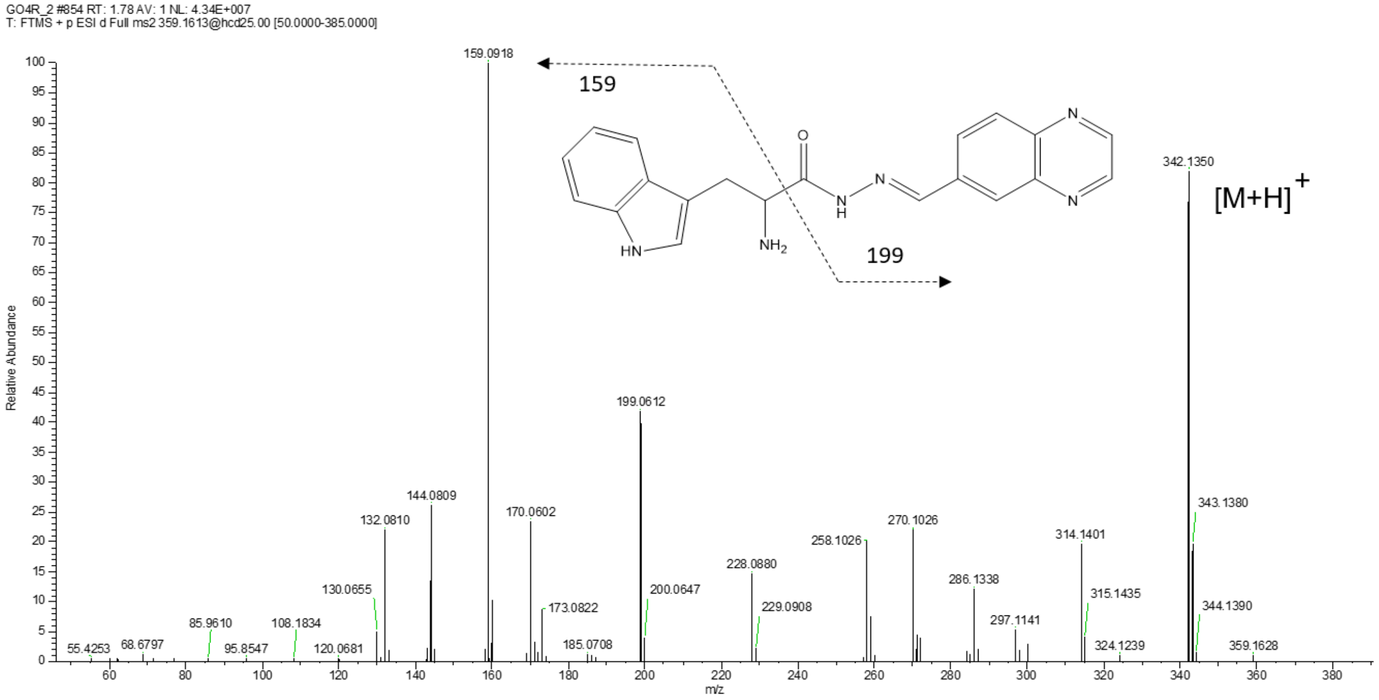
GO4R_2 #854 RT: 1.78 AV: 1 NL: 4.34E+007
T: FTMS + p ESI d Full ms2 359.1613@hcd25.00 [50.0000-385.0000]
Relative Abundance
m/z
159.0918
159
199
199.0612
342.1350
[M+H]+
343.1380
144.0809
132.0810
170.0602
130.0655
173.0822
200.0647
228.0880
229.0908
258.1026
270.1026
286.1338
297.1141
314.1401
315.1435
344.1390
324.1239
359.1628
185.0708
120.0681
108.1834
95.8547
85.9610
68.6797
55.4253
HN
NH2
O
N
H
N
N
N

A. R-G04

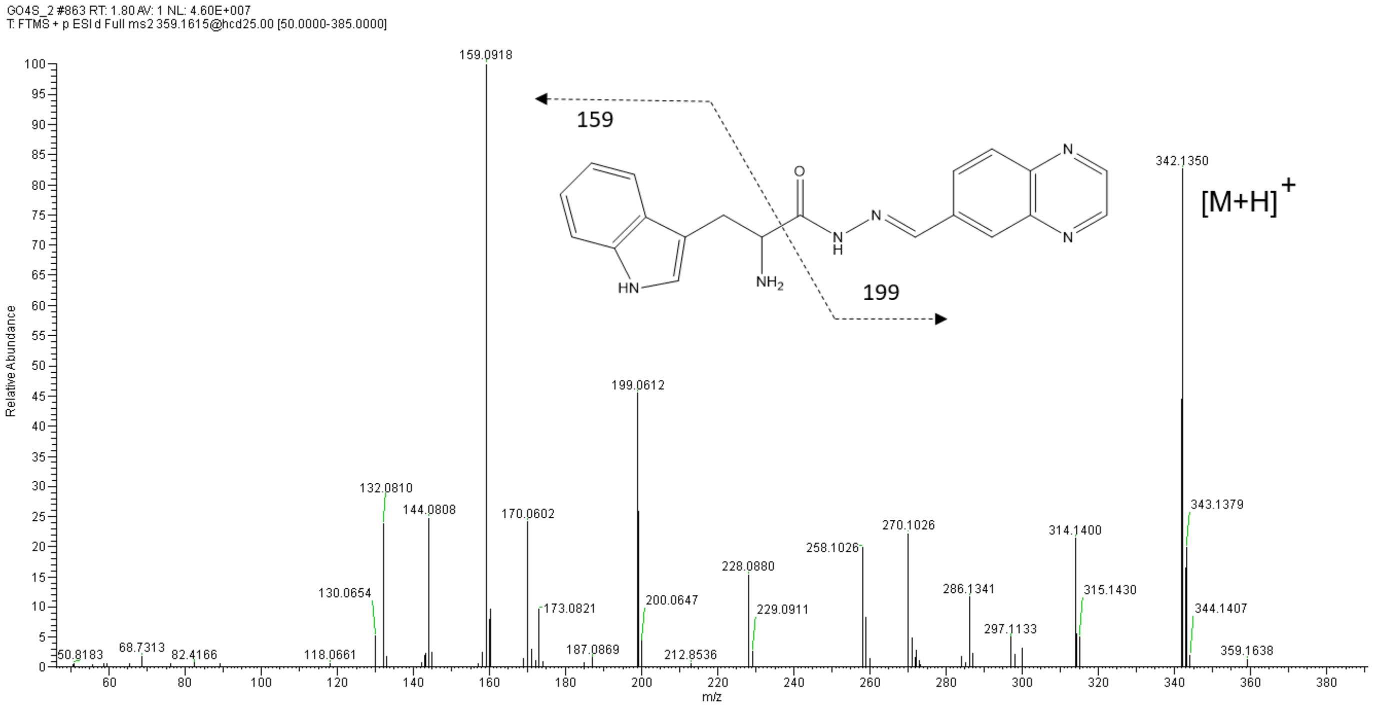

B. S-G04

Figure S6. Fragmentation pattern peak to confirm the 2 enantiomers, R-G04 and S-G04. (A-B) R-G04 and S-G04 in DMSO solution were dissolved into acetonitrile/water (1/1 v/v). 10 μL of the prepared solution was injected into Q ExactiveTM plus hybrid quadrupole-OrbitrapTM mass spectrometer interfaced with Vanquish UHPLC-HRMS (M represents the desired compound).
